# Dynamic metabolic modeling of heterotrophic and mixotrophic microalgal growth on fermentative wastes

**DOI:** 10.1371/journal.pcbi.1005590

**Published:** 2017-06-05

**Authors:** Caroline Baroukh, Violette Turon, Olivier Bernard

**Affiliations:** 1 INRA, UR0050 Laboratoire de Biotechnologie de l'Environnement, Narbonne, France; 2 INRIA, BIOCORE, Sophia-Antipolis, FRANCE; 3 UPMC, LOV, CNRS UMR 7093, Station Zoologique, Villefranche-sur-mer, FRANCE; EMBL-Heidelberg, GERMANY

## Abstract

Microalgae are promising microorganisms for the production of numerous molecules of interest, such as pigments, proteins or triglycerides that can be turned into biofuels. Heterotrophic or mixotrophic growth on fermentative wastes represents an interesting approach to achieving higher biomass concentrations, while reducing cost and improving the environmental footprint. Fermentative wastes generally consist of a blend of diverse molecules and it is thus crucial to understand microalgal metabolism in such conditions, where switching between substrates might occur. Metabolic modeling has proven to be an efficient tool for understanding metabolism and guiding the optimization of biomass or target molecule production. Here, we focused on the metabolism of *Chlorella sorokiniana* growing heterotrophically and mixotrophically on acetate and butyrate. The metabolism was represented by 172 metabolic reactions. The DRUM modeling framework with a mildly relaxed quasi-steady-state assumption was used to account for the switching between substrates and the presence of light. Nine experiments were used to calibrate the model and nine experiments for the validation. The model efficiently predicted the experimental data, including the transient behavior during heterotrophic, autotrophic, mixotrophic and diauxic growth. It shows that an accurate model of metabolism can now be constructed, even in dynamic conditions, with the presence of several carbon substrates. It also opens new perspectives for the heterotrophic and mixotrophic use of microalgae, especially for biofuel production from wastes.

## Author summary

Most existing metabolic modeling tools are not suitable for studying diauxic growth with dynamic substrate shifts. This paper describes a successful modeling of *Chlorella sorokiniana* metabolism, based on 172 reactions and validated by nine independent dynamic experiments (nine experiments were used for its calibration), in which microalgae were grown heterotrophically or mixotrophically on acetate and/or butyrate, in the light or dark. Such an extensive validation has not been performed before for microalgae. It was demonstrated that the model could be used to assess the flux dynamics in the cell. This innovative simulation tool was also used to derive original strategies to bypass the toxicity of some substrates using mixotrophic regimes.

## Introduction

Microalgae are unicellular eukaryote microorganisms that can grow autotrophically using light energy and CO_2_. Many species can also grow heterotrophically in darkness on various organic carbon sources, including glucose, or can combine heterotrophy and autotrophy for a mixotrophic growth [1]. Microalgae have been domesticated and used to synthesize many products with industrial applications, such as pharmaceutics or cosmetics (antioxidants, pigments, unsaturated long-chain fatty acids), agricultural products (food supplements, functional food, colorants) and animal feed (aquaculture, poultry or pig farming) [2]. They are also promising organisms for green chemistry (bioplastics), the environment (wastewater treatment, CO_2_ mitigation), and even energy production (biodiesel, bioethanol, hydrogen) [2].

Autotrophic growth of microalgae is limited by light distribution to all the cells, constraining the cell concentration to below 10 g/l (for the thinnest and most concentrated cultivation systems). Heterotrophic growth does not have this limiting factor and higher biomass density can be achieved [1], drastically reducing the harvesting costs. In addition, heterotrophic growth is usually faster, reducing the cultivation time [3]. However, industrial production of heterotrophic microalgae is hampered by the high economic and environmental costs of glucose, commonly used as a substrate. One solution is to use the waste from other processes, such as glycerol, acetate (ACE) or butyrate (BUTYR), which represent low cost carbon substrates. For instance, dark anaerobic fermentation produces an effluent mainly composed of acetate and butyrate [4]. However, some substrates in waste, such as butyrate, can be inhibitory [5]. Moreover, the successive metabolic switches between different substrates are not well understood and are likely to significantly affect growth. Therefore, this bioprocess still needs to be mastered and optimized to produce microalgae and extract the targeted byproducts on an industrial scale and at a competitive price, with consistent quality and in a sustainable way.

In this context, mathematical modeling of the metabolism has proven to be an efficient tool for optimizing growth and increasing the production of target molecules. To date, no models exist for heterotrophic microalgal metabolism dynamically switching between several substrates (S1 Table), including mixotrophic growth in light. So far, only static fluxes have been predicted under constant substrate consumption [6–9]. Representing the dynamic shifts for a blend of substrates typical of real wastewater is a major challenge, since some intracellular accumulation might occur, either during the transition between substrates, or due to the varying nature of the light. As a consequence, the quasi-steady state assumption (QSSA) required by most of the existing metabolic approaches may be an invalid hypothesis in this case [10]. The DRUM modeling framework recently proposed in [10] was used here to handle the non quasi-steady state (QSS). It allowed the development of a dynamic metabolic model for *Chlorella sorokiniana* grown on a single-substrate culture and a mixed-substrate (acetate and butyrate) culture, combined with various combinations of light. The model is thus designed to represent autotrophic, heterotrophic or mixotrophic modes under diauxic conditions. Our purpose is to propose a relatively generic model, instantiated and calibrated for *C*. *sorokiniana*. According to Baroukh et al. [11], such a generic model should be applicable to a wide range of microalga species.

## Model

### Experimental conditions

The goal of the experiments was to grow *Chlorella sorokiniana* on a synthetic medium mimicking the digestate composition produced by a dark fermenter processing organic waste. At this stage, the composition of the medium was kept simple, with only the two main organic components—acetate and butyrate [4]–to gain a clear understanding of their effects on microalgae growth. *Chlorella sorokiniana* was grown both in the dark and in the light (136 μE.m^-2^.s^-1^), in axenic conditions at 25°C and constant pH (6.5) in triplicate batches with different initial concentrations of acetate and butyrate (Table 1). Nitrogen (ammonium) and phosphorus were provided in non-limiting concentrations in order to focus solely on carbon metabolism. To ensure that no substrate was favored because of acclimation, the inoculum was grown autotrophically beforehand. See Turon et al. [5,12] for more details of the experimental protocols.

**Table 1:** List of the experimental conditions.

Experiments	Initial conditions		Data used for Estimation (E) or validation (V)
	Acetate (gC.L^-1^)	Butyrate (gC.L^-1^)	
Growth on acetate only	0.1	-	E
	0.25	-	V
	0.30	-	V
	0.5	-	V
	1	-	E
Growth on butyrate only	-	0.1	E
	-	0.25	V
	-	0.5	V
	-	1	V
Growth on acetate and butyrate mixtures	0.25	0.25	E
	0.25	0.5	V
	0.4	0.1	V
	0.5	0.9	E
	0.9	0.1	V
Autotrophic and mixotrophic growth	-	-	E
	0.3	-	E
	-	0.3	E
	0.3	0.3	E

### Metabolic network construction

A detailed description of the metabolic network reconstruction is provided in S1 File. Since *Chlorella sorokiniana* has not been sequenced yet, no genome-scale metabolic network (GSMN) reconstruction was possible. However, the core carbon and nitrogen metabolic networks in the GSMN of previously reconstructed microalgae species are relatively similar [13]. Thus, the conserved core metabolic network was used, containing the central metabolic pathways relevant to mixotrophy and heterotrophy: photosynthesis, glycolysis, pentose phosphate pathway, citric acid cycle, oxidative phosphorylation, and synthesis of chlorophyll, carbohydrates, amino acids and nucleotides. Species-specific pathways such as the synthesis of secondary metabolites were not represented, since these pathways were assumed to represent negligible fluxes compared to the main pathways and thus to have little impact on the metabolism. The reactions involved in macromolecule synthesis (proteins, lipids, DNA, RNA and biomass) were lumped into generic reactions. The growth-associated ATP maintenance (GAM) was replaced by the value experimentally measured by Boyle et al. (2009) for growth of *Chlamydomonas reinhardtii* on acetate. They observed 29.890 moles of ATP for 1000g of biomass. In our model, the biomass reaction yields 186g of biomass; the maintenance term is thus 5.56 moles of ATP per mol of biomass (details on how this value was computed are available in S1 File). A sensitivity analysis was carried out to assess the impact of this maintenance value on model accuracy (S1 Fig). Results showed that the optimal growth associated yield is 12.2mol ATP per mol of biomass, with [0–16.7 molATP/mol B] as the 10% interval confidence. The value of Boyle et al [6] falls in this interval, demonstrating that an error of this term in this range has a minor impact on model predictions. The non-growth ATP maintenance (NGAM) was assumed negligible. It may explain the slightly higher value of the optimal growth associated yield resulting from optimization, which might also compensate for NGAM.

### Definition and reduction of the sub-networks

**Fig 1:**
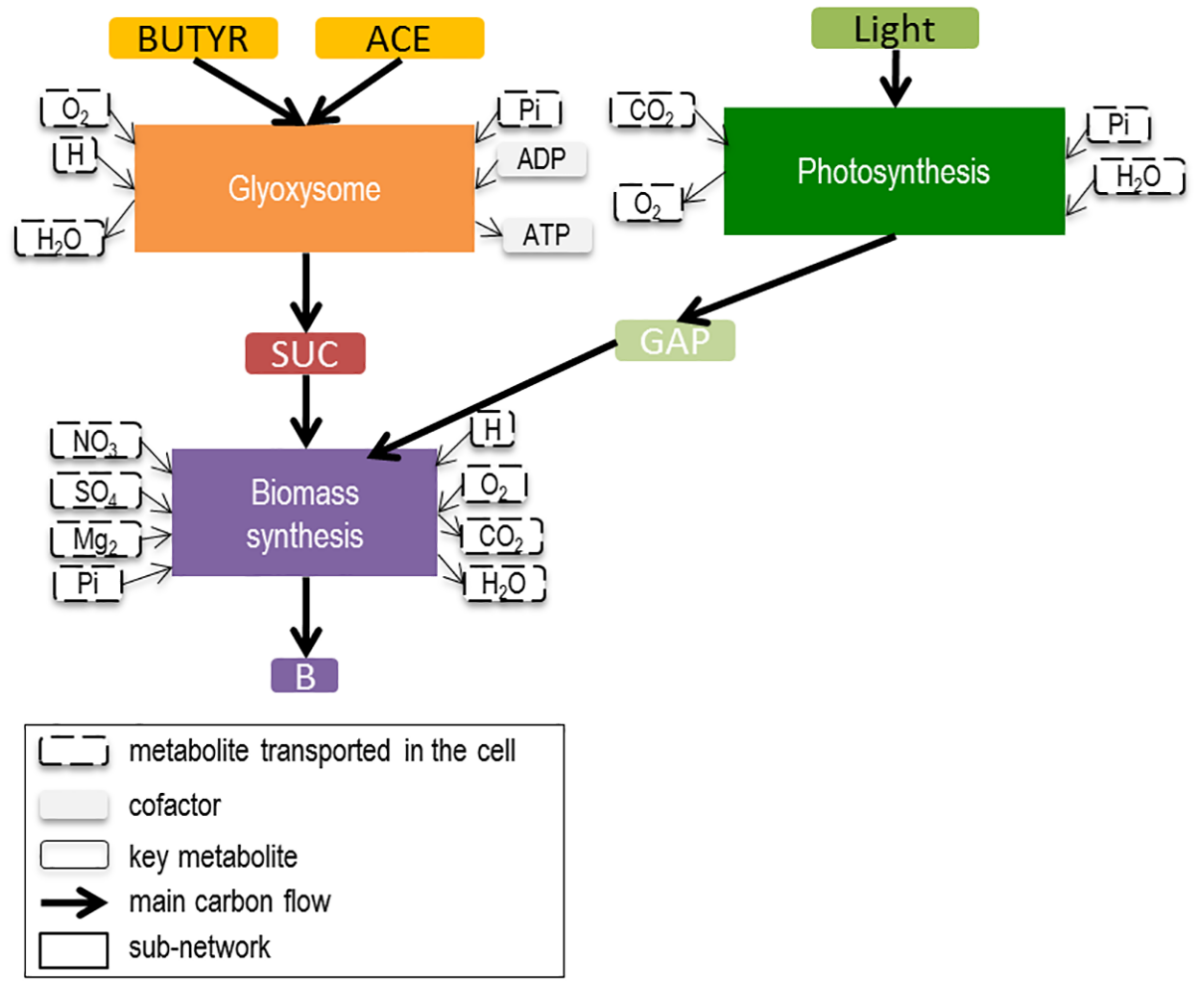
Central carbon metabolic network of a unicellular heterotrophic microalga decomposed into three sub-networks. Central carbon metabolic network is composed of photosynthesis, the glyoxysome, citric acid cycle, glycolysis, carbohydrate synthesis, pentose phosphate pathway, lipid synthesis, oxidative phosphorylation, protein, DNA, RNA, chlorophyll and biomass synthesis. During photosynthesis, inorganic carbon (CO_2_) is assimilated using light energy to produce a 3-carbon sugar glyceraldehyde 3-phosphate (GAP). In the glyoxysome, fatty acids (including acetate and butyrate) are degraded to Acetyl-CoA, which is then transformed to succinate (SUC) thanks to the glyoxylate cycle. SUC and GAP are then used as primary precursors to produce precursor metabolites and energy via the Tricarboxylic Acid (TCA) cycle for protein, DNA, RNA, carbohydrate and lipid synthesis.

Generally, metabolic modeling relies on the QSSA of the whole metabolic network, where intracellular metabolites cannot accumulate or be depleted [14]. The idea of the DRUM approach is to mildly relax this hypothesis, by splitting the metabolic network into a limited number of sub-networks [10], for each of which the QSS is assumed. The metabolites situated at the junction between the sub-networks, can therefore have dynamics of accumulation and depletion. The sub-networks are defined by metabolic functions and take into account cellular compartments. This assumption is supported by the idea that cell function and cell compartment are often associated with co-regulation and substrate channeling, which implies synchronicity of reactions and thus quasi-steady state for those reactions [10]. The idea is also to find a network splitting simple enough for explaining the experimental data, so as to avoid overfitting by postulating too many reactions kinetics [10]. Further details on the philosophy behind the network splitting and the DRUM framework are given in the discussion section of Baroukh et al. [10] (DRUM principles are also summarized in supplementary information (S1 File, section 8)). For representing the growth of *Chlorella sorokiniana* with different inorganic or organic carbon sources, the network was split taking into account the compartments of the cell with a global catabolic or anabolic function (Fig 1): *i)* the glyoxysome for acetate and butyrate assimilation, *ii)* the chloroplast for photosynthesis, and *iii)* the rest of the reactions for functional biomass production (synthesis of lipids, carbohydrates, proteins, DNA, RNA and chlorophyll). Several splitting were tested, particularly on the transported metabolites between the glyoxysome and the cytosol (which are not consistent in literature). The best fitting results were obtained with these three sub-networks. Apart from inorganic compounds, only succinate (SUC) and glyceraldehyde 3-phosphate (GAP) were intracellular metabolites (*A*) that potentially accumulate. The idea is that these metabolites, which shuttle between the compartments (respectively between the glyoxysome and the cytosol and between the chloroplast and the cytosol), and which act as intermediate between catabolism and anabolism, are the ones that could act as “buffers” inside the cell. Each sub-network was balanced in cofactors and in chemical elements (carbon, oxygen, nitrogen, phosphorus, sulfur).

Each sub-network was then reduced to macroscopic reactions (MRs) using elementary flux mode analysis [15]. To compute elementary flux modes (EFMs), the *efmtool* program was used [16]. For the three sub-networks, the EFM could be computed easily, since their total number was less than 1751 (it should be noted that an EFM analysis of the full network results in 5105 modes).

### Analysis of the glyoxysome sub-network

Glyoxysomes are specialized peroxisomes found in plants or microalgae [8], in which fatty acids (including acetate and butyrate) can be used as a source of energy and carbon for growth when photosynthesis is not active. Fatty acids are hydrolyzed to acetyl-CoA, and then transformed into succinate via the glyoxylate cycle. Succinate can then be transformed into a variety of macromolecules for biomass growth, through combinations of other metabolic processes taking part in other compartments of the cell.

Reduction of the glyoxysome sub-network yielded two MRs, one for each substrate (Table 2).

**Table 2:** Definition and reduction of sub-networks formed from metabolic reactions of *Chlorella sorokiniana* for heterotrophic and mixotrophic growth.

Sub-network	Macroscopic reactions	Kinetics
Acetate & Butyrate assimilation	3.5 H + 2 ACE + 0.5 O2 —> SUC + 1.5 H2O (MR1)	$\alpha_{MR1}\;=\;k_{MR1}*\frac{ACE}{Ks_{MR1}+ACE}$
	7 H + 1.5 O2 + 1 BUTYR —> 1 SUC + 5 H2O (MR2)	$\alpha_{MR2}\;=\frac{k_{MR2}*BUTYR}{BUTYR+\frac{k_{MR2}}{\beta_{MR2}}*{\left(\frac{BUTYR}{Sopt_{MR2}}-1\right)}^{2}}*\frac{k_{D}}{ACE+k_{D}}$
Photosynthesis	24 Light + 3 CO2 + 2 H2O + 1 Pi—> 1 GAP + 3 O2 (MR3)	$\alpha_{MR3}=\frac{\gamma_{MR3.}\left(1-e^{\beta_{MR3}.B}\right)}{\beta_{MR3}.B}$
Biomass synthesis	7.30239 H + 4.61237 O2 + 4.14597 SUC + 0.984915 NH4 + 0.1216 Pi + 0.02169 SO4 + 0.0101 Mg2 —> 1 Biomass + 7.04167 H2O + 8.06249 CO2 (MR4)	*α*_*MR*4_ = *k*_*MR*4 * *SUC*_
	4.14597 GAP + 2.53938 O2 + 0.984916 NH4 + 0.02169 SO4 + 0.0101 Mg2 —> 0.989545 H + 1 Biomass + 2.8957 H2O + 3.91652 CO2 + 4.02437 Pi (MR5)	*α*_*MR*5_ = *k*_*MR*5 * *GAP*_

Each sub-network was decomposed into a set of macroscopic reactions using elementary flux mode analysis. Lists of reactions, incoming and outgoing metabolites for each sub-network are available in S1 File section 5.

### Analysis of the photosynthesis sub-network

Photosynthesis supports the generation of cell energy in phototrophic organisms and contributes to the incorporation of inorganic carbon. The process takes place in the chloroplast and consists of two steps commonly known as the light and dark reactions. The light reaction consists of the generation of cell energy (ATP, NADPH) from water and photons, producing oxygen. Thanks to the energy of the light reaction, the dark step reactions incorporate carbon dioxide through the Calvin cycle producing a 3-carbon sugar (3-phosphoglycerate, or 3PG). Then, 3PG is transformed into glyceraldehyde 3-phosphate (GAP) and transported to the cell cytosol.

Elementary flux mode analysis of this sub-network yielded only one Elementary Flux Mode (EFM) (Table 2), associated with one macroscopic reaction (MR3). The stoichiometry of the derived macroscopic reaction is in agreement with the literature: a quota of 8 photons are needed per carbon incorporated [17].

### Analysis of the functional biomass synthesis sub-network

The synthesis reactions of lipids, proteins, DNA, RNA, chlorophyll and carbohydrates were grouped into a functional biomass synthesis sub-network and assumed to be in QSS. This sub-network includes glycolysis, TCA cycle, oxidative phosphorylation, pentose phosphate pathway, nitrogen and sulfur assimilation, carbohydrate synthesis, lipid synthesis, amino acid synthesis and nucleotide synthesis.

The reduction of this sub-network yielded 1748 EFMs, which is reasonable given the number of involved reactions (143), and much lower than the number of modes for the full network (5105). Most of these EFMs (1618) yielded biomass, while the others correspond to futile cycles. In terms of carbon, once normalized by unit of biomass synthesis flux, the 1618 MRs deduced from the EFMs only differed in their consumption of SUC and GAP and their production of CO_2_ (S2 and S3 Figs). As in Flux Balance Analysis [18], we assumed that the cell maximized biomass growth, and hence minimized carbon loss when synthesizing biomass from each substrate. The elementary flux modes with the best SUC/CO_2_ yield and GAP/CO_2_ yield were thus chosen (Table 2). The resulting MR consumes SUC or GAP and NH_4_ for carbon and nitrogen sources, SO_4_ and Mg for protein and chlorophyll synthesis and O_2_ for ATP synthesis through oxidative phosphorylation.

### Dynamic modeling

**Fig 2:**
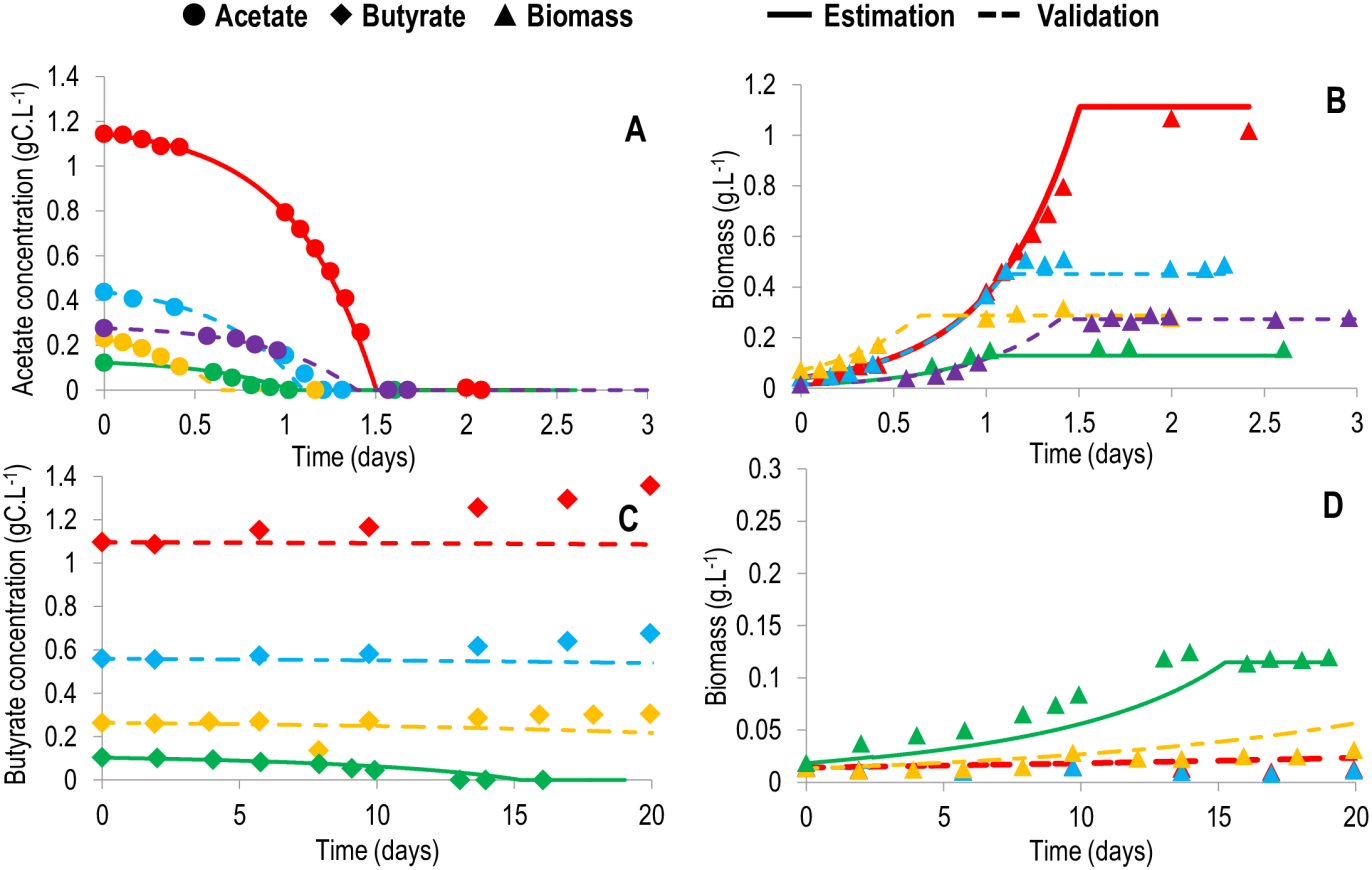
Comparison between the model and experimental data for *Chlorella sorokiniana* heterotrophic growth on acetate or butyrate. Simulations are represented by full lines (conditions used for calibration) or dashed lines (conditions used for validation). Experimental results are represented by large dots, triangles or diamonds. Red: 1 gC.L^-1^; blue: 0.5 gC.L^-1^; purple: 0.3 gC.L^-1^; yellow: 0.25 gC.L^-1^; green: 0.1 gC.L^-1^. A. Acetate concentration (gC.L^-1^) for acetate growth. B. Biomass concentration (g.L^-1^) in acetate growing conditions. C. Butyrate concentration (gC.L^-1^) for butyrate growth. D. Biomass concentration (g.L^-1^) in butyrate growing conditions. Thanks to the fitting quality, each of the experimental triplicates could be appropriately fit accounting for the slight variations in the initial conditions. Only one of the triplicates per experimental condition is represented here, but the simulations for all triplicates are available in S4 Fig.

At this stage, the macroscopic kinetics of the MRs must be determined in order to simulate the metabolic dynamics [10]. We assumed that only the carbon substrates of each MR were limiting, playing thus a role in the kinetics. Michaelis-Menten kinetics was used for acetate consumption (Table 2), since experimental data showed no growth inhibition on acetate. Haldane kinetics was chosen for the butyrate consumption reaction (Table 2), since experimental data showed that butyrate inhibited biomass growth (growth only possible with maximum 0.1 gC.L^-1^ (Fig 2C) in butyrate-only experiments).

**Fig 3:**
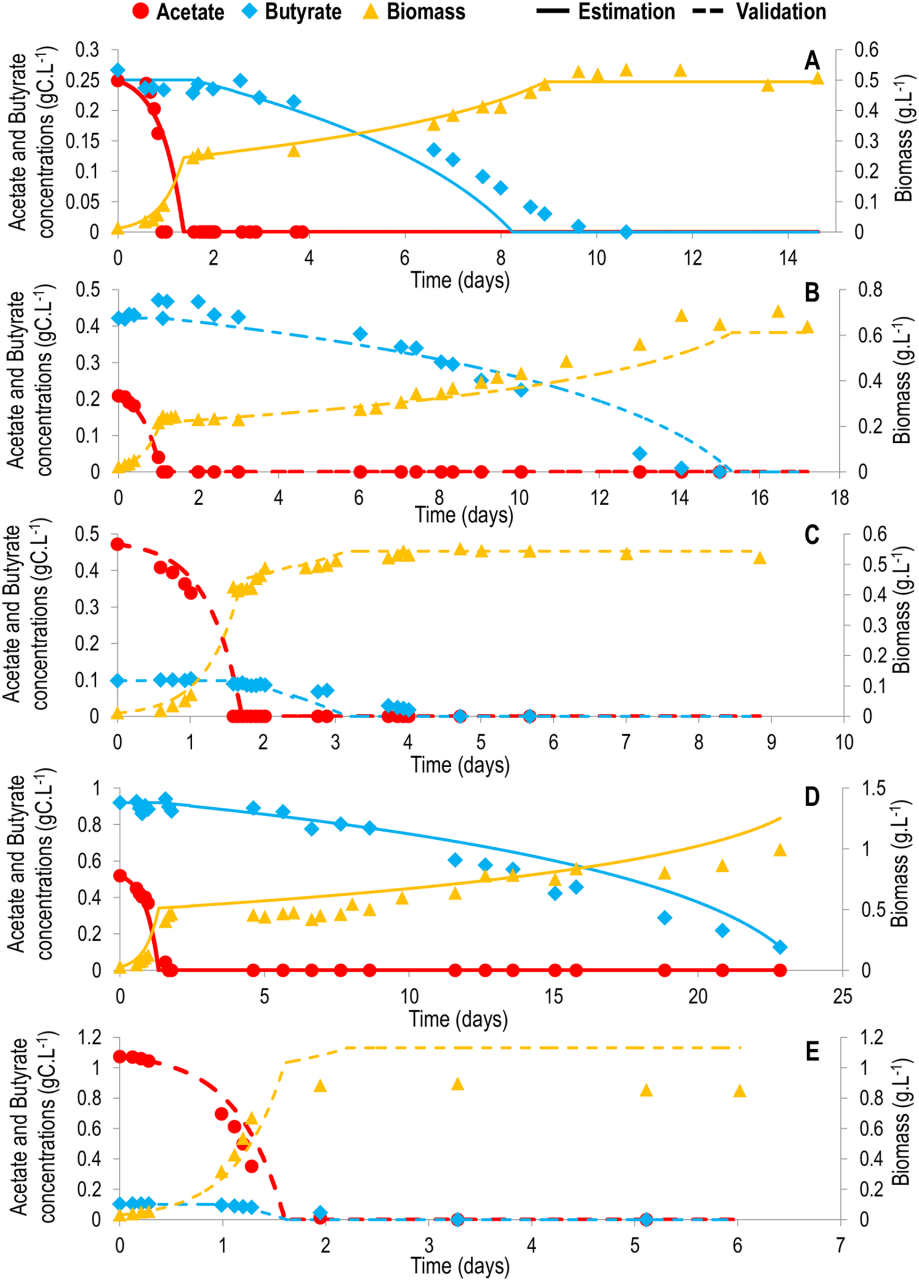
Comparison between the model and experimental data for *Chlorella sorokiniana* heterotrophic growth mixtures of acetate and butyrate. Simulations are represented by full lines (conditions used for calibration) or dashed lines (conditions used for validation). Experimental results are represented by large dots, triangles or diamonds. Red: acetate (gC.L^-1^); blue: butyrate (gC.L^-1^); yellow: biomass (g.L^-1^). A. Growth on 0.25 gC.L^-1^ acetate and 0.25 gC.L^-1^ butyrate. B. Growth on 0.25 gC.L^-1^ acetate and 0.5 gC.L^-1^ butyrate. C. Growth on 0.4 gC.L^-1^ acetate and 0.1 gC.L^-1^ butyrate. D. Growth on 0.5 gC.L^-1^ acetate and 0.9 gC.L^-1^ butyrate. E. Growth on 0.9 gC.L^-1^ acetate and 0.1 gC.L^-1^ butyrate. Only one of the experimental triplicates is represented here. The simulations for all triplicates are available in S5 Fig.

Biomass growth clearly exhibited a diauxic growth for the mixed substrate conditions: acetate was entirely consumed before the butyrate concentration started to decrease (Fig 3). This diauxic growth is probably the result of transcriptional regulations taking place inside the cell. DRUM framework, can explicitly describe such regulations via an appropriate choice of the kinetics in connection with metabolite accumulation. However, to the best of our knowledge, the transcriptional regulations responsible for this diauxic growth are not known. It is thus premature to propose an explicit mathematical expression for representing this phenomenon; a general kinetic expression for diauxic growth, which implies that regulation is performed by acetate directly on the cell transporter of butyrate, was thus chosen. An inhibitory term of acetate concentration on butyrate consumption kinetics was included in the butyrate uptake kinetics (Table 2). Linear kinetics depending on the mean light intensity in the reactor was chosen to represent photosynthesis (Table 2). The mean light intensity was computed using a Beer-Lambert law (S1 File). Linear kinetics with respect to the carbon substrate were chosen for biomass synthesis (Table 2).

Finally, the dynamics of the 172 fluxes in the metabolism can be derived from a system of 14 differential equations comprising 14 metabolites and 5 macroscopic reactions representing 3 compartments:

$$\frac{dM^{\prime }}{dt}=\frac{d\left(\begin{array}{c} S\\ A\\ B \end{array}\right)}{dt}=K^{\prime }.\alpha .B$$

where *M’* is the vector of metabolites (14x1) composed of substrate *S*, metabolites susceptible to accumulate *A* (SUC and GAP) and functional biomass *B*; *K’* is the reduced stoichiometric matrix (14x5) and α is the kinetics vector (5x1) (Table 2, S6 Fig). The way all the metabolic fluxes are computed from *K’* and α is recalled in S1 File. Total biomass *X* (g.L^-1^) is computed thanks to a mass balance on the cell:

$$X\left(t\right)=\underset{A}{\sum }M_{A}.A\left(t\right)+M_{B}.B\left(t\right)$$

with *M*_*A*_ and *M*_*B*_ the molar masses of metabolites *A* and *B* (for further details, see S1 File section 5).

The dynamic model has 10 degrees of freedom, and each degree is represented by a parameter that needs to be calibrated. To estimate the parameters, we minimized the squared-error between simulation and experimental measurements using the Nelder-Mead algorithm [19] (function *fminsearch* in Scilab^®^). To reduce the risk of local minima, several optimizations were performed with random initial parameters. Nine experiments were used to estimate the parameters (Table 1); the nine remaining experiments were reserved to assess the validity of the model (Table 1). Results of the parameter identification are presented in Table 3.

**Table 3:** Parameters obtained by calibration of the model.

Parameters	Value with DRUM	Definition
*k*_*MR*1_	3.79*10^−1^ M.h^-1^.M B^-1^	Maximal acetate assimilation rate
*Ks*_*MR*1_	5.52*10^−5^ M	Half-saturation constant for acetate assimilation
*k*_*MR*2_	3.61*10^−2^ M.h^-1^.M B^-1^	Maximal butyrate assimilation rate
*β*_*MR*2_	2.58*10^5^ h^-1^.M B^-1^	Butyrate inhibition constant
*Sopt*_*MR*2_	1.93*10^−5^ M	Optimal concentration for butyrate assimilation
*K*_*D*_	5.39*10^−10^ M	Diauxic constant
*γ*_*MR*3_	2.62*10^−1^ M.h^-1^.M B^-1^	Photosynthesis kinetic parameter
*β*_*MR*3_	2.48*10^3^ M B^-1^	Light attenuation parameter
*k*_*MR*4_	2.37*10^5^ h^-1^.M B^-1^	GAP biomass synthesis kinetic parameter
*k*_*MR*5_	2.83*10 h^-1^.M B^-1^	SUC biomass synthesis kinetic parameter

## Results/Discussion

### Macroscopic scale simulation

**Fig 4:**
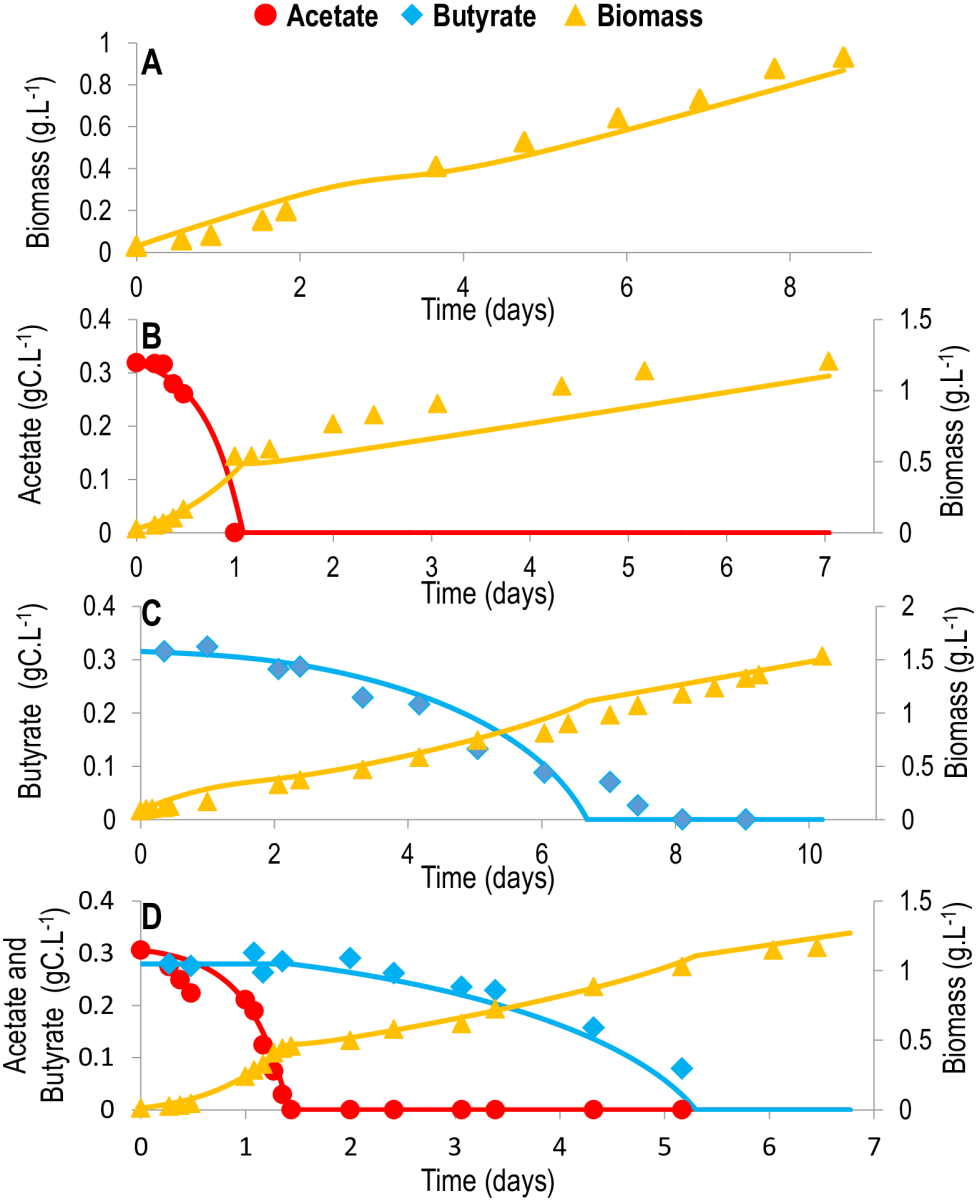
Comparison between the model and experimental data for *Chlorella sorokiniana* mixotrophic and autotrophic growth. Simulations are represented by full lines (conditions used for calibration). Experimental results are represented by large dots, diamonds or triangles. Red: acetate; blue: butyrate; yellow: biomass. A. Autotrophic growth. B. Mixotrophic growth with 0.3 gC.L^-1^ acetate C. Mixotrophic growth with 0.3 gC.L^-1^ butyrate. D. Mixotrophic growth with 0.3 gC.L^-1^ acetate and 0.3 gC.L^-1^ butyrate. Only one of the experimental triplicates is represented here. The simulations for all triplicates are available in S7 Fig.

The model simulation accurately reproduces experimental data, even for the validation data sets that were not used for calibration (Figs 2–4). The diauxic growth is particularly well represented (Figs 3 and 4D), and the transient behavior, together with the final biomass, is correctly predicted (Figs 2–4), showing that the biomass yields obtained from the metabolic network are accurate. Indeed, one of the advantages of metabolic modeling [20] is the prediction of biomass yields supported by the stoichiometry of the metabolic network. Here, the predicted conversion yield of acetate and butyrate to biomass is 0.514 grams of carbon biomass per gram of carbon in the incoming substrate. This yield contributes to correct prediction of the biomass for both acetate (Fig 2B) and butyrate (Fig 2D), thus validating the approach. Interestingly, the yields are identical between the two substrates. A possible explanation is the fact that more ATP is required for the transport of butyrate into the cell than for acetate, thus balancing the ATP created when converting butyrate and acetate to succinate.

The set of kinetic parameters matches both the single-substrate culture and the mixed-substrate culture. This implies that butyrate has no impact on acetate growth rate. However, the inverse is not true, since the acetate concentration at which butyrate consumption starts (*k*_*D*_) is very low (5.39*10^−10^ M), illustrating the strong diauxic growth that occurs. Even the smallest amount of acetate inhibited butyrate uptake. The maximum acetate uptake rate was higher than the maximum butyrate uptake rate by nearly 15 fold, reflecting the preference of *Chlorella sorokiniana* for acetate. The non-inhibiting butyrate concentration (*Sopt*_*MR*2_) was very low (1.93*10^−5^ M), which highlights the strong inhibition of butyrate in the medium on its uptake. It also explains why, in the butyrate-only experiments, no biomass growth was observed for butyrate concentrations above 0.1 g.L^-1^ (Fig 2D).

In addition to substrates and biomass concentrations, light evolution inside the culture vessel was computed. During the first few days, the average light intensity decreases until equilibrium is reached around 16 μE.m^-2^.s^-1^ (S1 File section 4, S8 Fig). It represents 11.7% of the incident light and is in agreement with the literature [21]. Interestingly, equilibrium is reached faster for mixotrophic growth, particularly on acetate, which supports fast heterotrophic growth (S8 Fig). In addition, the photosynthetic quotient for autotrophic growth varies between 1.0 and 1.16, matching the typical range of 1.0–1.8 for microalgae [6].

### Intracellular scale simulation

**Fig 5:**
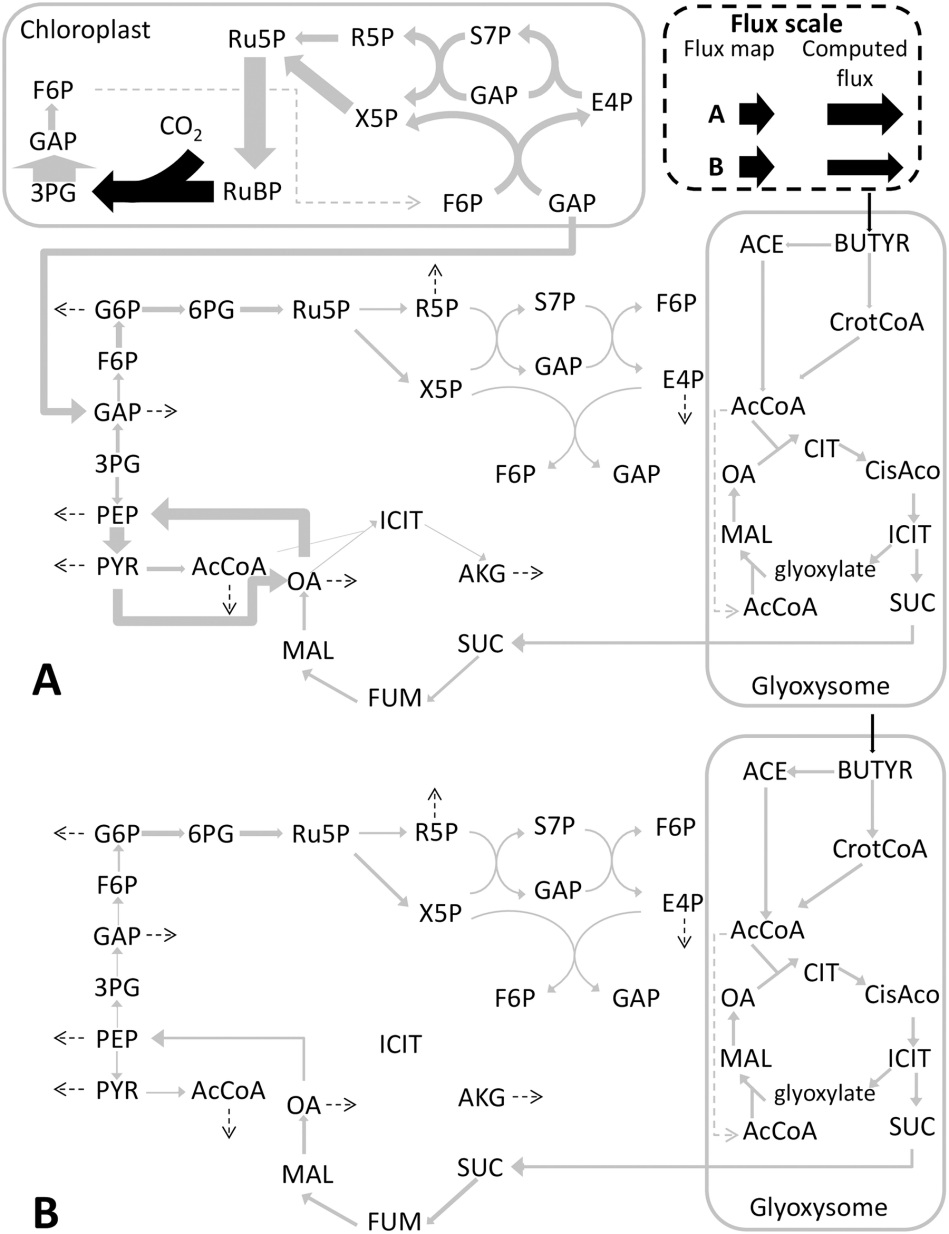
Flux maps for mixotrophic and heterotrophic growth of *Chlorella sorokiniana* on butyrate. Fluxes are normalized by unit of biomass. Dashed arrows indicate fluxes related to biomass formation. Metabolic fluxes vary greatly according to substrates and growth modes. The scale for converting metabolic fluxes into arrows width is presented for each case. A. Mixotrophic growth on 0.3 g.L^-1^ butyrate. Flux maps computed at time = 5.0 days. B. Heterotrophic growth on 0.1g.L^-1^ butyrate. Flux maps computed at time = 8.1 days. Flux maps on other substrates are available in S9 Fig.

The predicted metabolic fluxes (Fig 5, S9 Fig) are in accordance with previous studies [11]. Autotrophy (S9C Fig) is characterized by high fluxes in the photosynthetic pathways, which convert light and CO_2_ to GAP. Beyond these pathways, fluxes drop considerably in terms of absolute magnitude. Upper glycolysis is in the gluconeogenic direction to produce the carbohydrate and sugar precursor metabolites (Glucose 6-phosphate (G6P), Ribose 5-phosphate (R5P), Erythrose 4-phosphate (E4P)) necessary for growth. In the heterotrophic mode, fluxes are more homogenous among reactions (Fig 5B, S9A Fig). Acetate and butyrate are converted to acetyl-CoA in the glyoxysome (Fig 5B, S9D Fig). Acetyl-CoA is then converted into succinate by the glyoxylate cycle and injected in the TCA cycle. Upper glycolysis also goes in the gluconeogenic direction to produce carbohydrate and sugar precursors. This can be achieved thanks to the anaplerotic reactions that convert oxaloacetate to phosphoenolpyruvate (PEP). Mixotrophy is a mixed combination of the autotrophic and heterotrophic modes (Fig 5A, S9A Fig). For mixotrophic growth on acetate, heterotrophic metabolism is dominant, whereas autotrophic metabolism is dominant for mixotrophic growth on butyrate. This is due to the fact that autotrophic growth is slower than growth on acetate but faster than growth on butyrate.

### Avoiding the inhibitory effect of butyrate

**Fig 6:**
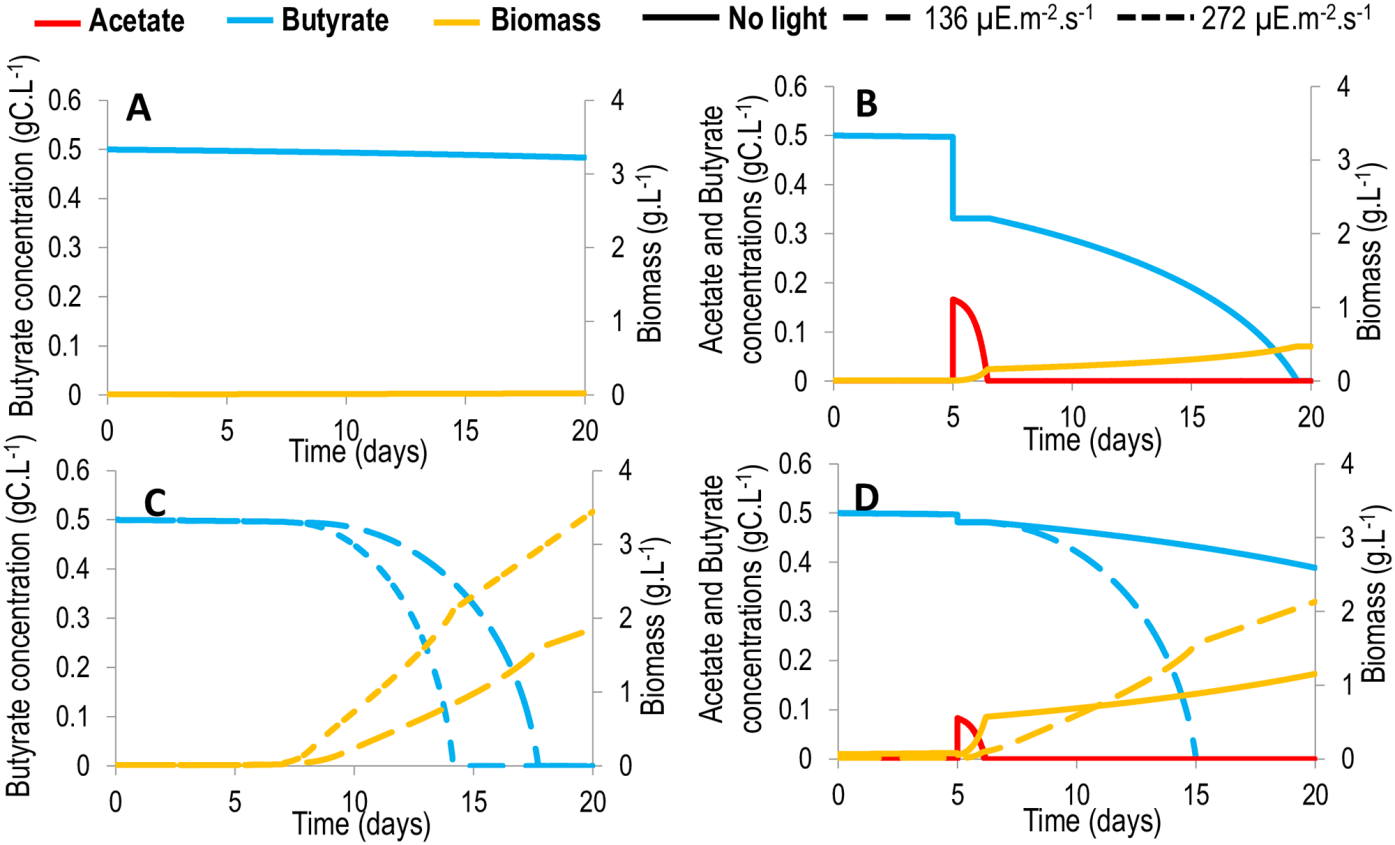
Disinhibition of butyrate by addition of acetate, light and a mix of acetate/butyrate due to the biomass effect. A larger biomass implies a decrease in butyrate inhibition on growth. Biomass can be increased by addition of acetate (B), light (C) and/or a mix of acetate and butyrate (D). Red: acetate; blue: butyrate; yellow: biomass. A. Normal conditions, without any additions. B. Addition of acetate (0.5 gC.L^-1^, volume half of culture volume) in the medium at day 5. C: Addition of light at day 5. Full lines: incident light intensity of 136 μE.m^-2^.s^-1^. Dashed line: incident light intensity of 272 μE.m^-2^.s^-1^. D. Addition of a mix of acetate (0.25 gC.L^-1^) and butyrate (0.45 gC.L^-1^) (volume half of culture volume) representative of a fermentative waste (4). Full lines: without light. Dashed lines: with light.

Interestingly, in agreement with the data, the model did not predict any growth on butyrate above 0.1 gC.L^-1^, and at the same time successfully forecasted growth on 0.9 gC.L^-1^ butyrate in mixed substrate conditions (Fig 3E) and on 0.3 gC.L^-1^ butyrate in mixotrophic conditions. Indeed, in these conditions, the first-stage growth on acetate and/or light produces enough biomass to finally consume such an inhibiting quantity of butyrate. The substrate to biomass (S/X) ratio is known to be a key process parameter for overcoming the inhibitory effects of the substrate [22]. The model therefore represents a tool to compute and optimize the amount of co-substrate that must be added to overcome the inhibition and consume the butyrate. Different strategies could be tested to achieve a low S/X ratio and accelerate butyrate consumption. The simplest approach would involve adding a non-inhibiting substrate in order to reduce the amount of inhibitory substrate per unit biomass. For example, the addition of 0.5 gC.L^-1^ of acetate for a volume equal to half of the culture volume has been found to eventually lead to the consumption of 0.5 gC.L^-1^ of butyrate in 14 days (Fig 6B), which would not have been possible otherwise (Fig 6A). However, in general, such pure substrate is not available. We therefore simulated the addition of a mix of acetate and butyrate in proportions that are representative of fermentative digestate [4], for a volume equal to half of the culture volume. On the one hand, the acetate contained in the waste stimulated growth, but since it is associated with addition of butyrate, it also increased inhibition. Simulations show that the inhibition is overcome, but does not lead to the total consumption of butyrate within 15 days (Fig 6D). Furthermore, the mixotrophic potential can be exploited: autotrophic growth can be enhanced by illumination in order to ultimately dilute the inhibitory substrates. Illuminating the algae at an incident intensity of 136 μE.m^-2^.s^-1^ leads to the consumption of the same quantity of butyrate in 13 days, and this delay can be reduced to 9 days using a light intensity of 272 μE.m^-2^.s^-1^ (Fig 6C). Finally, if light is provided at the same time as the addition of fermentative digestate (for a volume equal to half of the culture volume), inhibition can be overcome after 10 days (Fig 6D).

### Assessing the time constants of the metabolism

The advantage of the DRUM approach is its ability to account for the accumulation of some intracellular metabolites and thus to characterize the time to reach steady state. It can also determine more quantitatively the time scales of flux variations in the cell than earlier frameworks. This analysis was applied to SUC and GAP, which are, in our model, the intermediate accumulating metabolites.

Interestingly, SUC actually hardly accumulates in the simulations and rapidly achieves a QSS (S10 Fig) where its concentration evolves slowly compared to the other variables in the system (substrate consumption, biomass formation). We developed an algorithm to automatically detect the time needed to reach QSS (*t*_*QSS*_). In the experimental conditions of this study, approximately 3 minutes were necessary for succinate to achieve QSS (S2 File) thanks to a higher biomass synthesis rate (via a high *k*_*MR*4_) compared to the substrate assimilation rate, implying that succinate is immediately consumed once it is synthesized from butyrate or acetate. A sensitivity analysis on the parameter *k*_*MR*4_ revealed that the confidence interval of *t*_*QSS*_ was [0.6; 34] minutes (model error less than 5% of the minimal error) (S1 File section 8, S2 File). After the brief transient succinate step, the QSSA for heterotrophic growth on butyrate and acetate is valid. Therefore, the macroscopic model can be reduced further, by merging reaction MR4 with reactions MR1 and MR2 (S1 File section 6). The same kinetic parameters can be used for simulation, and the fit is nearly identical (increase of 0.6% of the error). As a consequence, results considering QSSA are very close to the ones based on DRUM.

GAP, in contrast to SUC, does not reach a QSS rapidly (S11 Fig). First, GAP accumulates at high light intensities, reaching a maximum when average light intensity is approximately 60 μE.m^-2^.s^-1^. Then, it is consumed at low light intensities, reaching a QSS when average light intensity reaches a steady state at 16 μE.m^-2^.s^-1^ (S1 File section 8). This suggests that microalgal metabolism in autotrophic and mixotrophic modes only reaches a QSS when average light is constant in the culture media, meaning that growth has ceased. This behavior is similar to that of microalgae grown in day/night cycles [10,11], involving accumulation of carbon-reserve metabolites (carbohydrates, lipids) during the day, when the light is intense enough, and re-consumption during the night or at the beginning and end of the day, when light intensity is low. Here, the carbon reserve metabolite is GAP, because only GAP accumulated in the model. Nevertheless, it is probable that carbohydrates and/or lipids also accumulate. Further experiments are required to validate these results more extensively and to determine which carbon-only metabolite is stored inside the cell.

To confirm these results, a Macroscopic Bioreaction Model of the system [23], relying on the QSSA assumption, was developed (see S1 File section 10 for details on the methodology). Without accumulation of SUC, the model error was almost unchanged (0.06% increase of the error). But without the possibility for GAP to accumulate, a 40% increase in the error is observed. This confirms our finding that GAP do accumulate inside the cell at high light intensities to be consumed later at lower light intensities. It is also interesting to note that the MBM approach is sufficient and produces accurate results, for applications in heterotrophy only cultures, without the need for accumulating metabolites.

### Conclusions

The dynamic metabolic model developed for the heterotrophic, mixotrophic and autotrophic growth of *Chlorella sorokiniana* on acetate and butyrate achieved a so far unequalled accuracy. The model efficiently fits the dynamic experimental data and correctly predicts the biomass yields for a broad range of experimental conditions. This new powerful simulation tool provides new insight into the mixotrophic microalgal process, and allows us to explore the different possibilities to overcome the inhibition induced by some of the substrates, in particular by adjusting the mixotrophic regimes. The model also highlights the dynamics of some internal compounds, especially under an auto- or mixotrophic regime, while light intensity is slowly affected by an increase in self-shading. As a consequence, the model shows that QSSA is not valid for mixotrophic growth as long as the light is variable in the culture medium. In the future, the model should be extended further in order to handle mixotrophic behavior under periodic light/dark cycles.

## Supporting information

S1 File Detailed metabolic network reconstruction process of *Chlorella Sorokiniana*; list of sub-networks; determination of the photosynthesis kinetic; determination of total biomass; construction of the derived macroscopic model for heterotrophic growth; algorithm construction for automatic detection of QSS; detailed summary of DRUM framework; computation of model prediction error; construction of macroscopic bioreaction model. (DOCX)

S2 File Estimation of the time needed to reach QSS for all experimental data sets and several *k*_*MR*4_ values. (XLSX)

S3 File Metabolic network at the SBML format. (SBML)

S1 Table Comparison of existing microalgal models representing heterotrophic and/or mixotrophic growth on acetate or butyrate. (DOCX)

S1 Fig Impact of growth-associated ATP maintenance (GAM) on model accuracy. The molecular weight of biomass (B) is 186g/mol. Formula of the error criterion is given in S1 File section 8.
(TIFF)

S2 Fig Projection of elementary flux modes obtained from the biomass synthesis sub-network in the SUC/CO2 yield space. (TIFF)

S3 Fig Projection of elementary flux modes obtained from the biomass synthesis sub-network in the GAP/CO2 yield space. (TIFF)

S4 Fig Comparison between the model and experimental data for *Chlorella sorokiniana* heterotrophic growth on acetate or butyrate for all triplicates. Simulations are represented by full lines (conditions used for calibration) or dashed lines (conditions used for validation). Experimental results are represented by large dots, triangles or diamonds. Shades of red: 1 gC.L^-1^; shades of blue: 0.5 gC.L^-1^; shade of purple: 0.3 gC.L^-1^; shade of yellow: 0.25 gC.L^-1^; shade of green: 0.1 gC.L^-1^. A. Acetate concentration (gC.L^-1^) for acetate growth. B. Biomass concentration (g.L^-1^) in acetate growing conditions. C. Butyrate concentration (gC.L^-1^) for butyrate growth. D. Biomass concentration (g.L^-1^) in butyrate growing conditions.
(TIFF)

S5 Fig Comparison between the model and experimental data for *Chlorella sorokiniana* heterotrophic growth mixtures of acetate and butyrate for all triplicates. Simulations are represented by full lines (conditions used for calibration) or dashed lines (conditions used for validation). Experimental results are represented by large dots, triangles or diamonds. Shades of red: acetate (gC.L^-1^); shades of blue: butyrate (gC.L^-1^); shades of yellow: biomass (g.L^-1^). A. Growth on 0.25 gC.L^-1^ acetate and 0.25 gC.L^-1^ butyrate. B. Growth on 0.25 gC.L^-1^ acetate and 0.5 gC.L^-1^ butyrate. C. Growth on 0.4 gC.L^-1^ acetate and 0.1 gC.L^-1^ butyrate. D. Growth on 0.5 gC.L^-1^ acetate and 0.9 gC.L^-1^ butyrate. E. Growth on 0.9 gC.L^-1^ acetate and 0.1 gC.L^-1^ butyrate.
(TIFF)

S6 Fig Stoichiometric matrix of the reduced model. (TIFF)

S7 Fig Comparison between the model and experimental data for *Chlorella sorokiniana* mixotrophic and autotrophic growth. Simulations are represented by full lines (conditions used for calibration). Experimental results are represented by large dots, diamonds or triangles. Shades of red: acetate; shades of blue: butyrate; shades of yellow: biomass. A. Autotrophic growth. B. Mixotrophic growth with 0.3 gC.L^-1^ acetate C. Mixotrophic growth with 0.3 gC.L^-1^ butyrate. D. Mixotrophic growth with 0.3 gC.L^-1^ acetate and 0.3 gC.L^-1^ butyrate.
(TIFF)

S8 Fig Evolution of average light intensity in the culture system. Green: Autotrophy, Red: Mixotrophy on acetate, Blue: mixotrophy on butyrate, Yellow: mixotrophy on acetate and butyrate.
(TIFF)

S9 Fig Flux maps of autotrophic, mixotrophic and heterotrophic growth of *Chlorella sorokiniana* on acetate and butyrate. Fluxes are normalized by unit of biomass. Dashed arrows indicate fluxes related to biomass formation. Metabolic fluxes vary greatly according to substrates and growth modes. The scale for converting metabolic fluxes into arrows width is presented for each case. A. Mixotrophic growth on 0.3g.L^-1^ acetate. Flux maps computed at time = 0.7 days. B. Mixotrophic growth on 0.3g.L^-1^ butyrate. Flux maps computed at time = 5.0 days. C. Autotrophic growth. Flux maps computed at time = 5.0 days. D. Heterotrophic growth on 0.3g.L^-1^ acetate. Flux maps computed at time = 1.0 days. E. Heterotrophic growth on 0.1g.L^-1^ butyrate. Flux maps computed at time = 8.1 days.
(TIFF)

S10 Fig Evolution of succinate in mixotrophic and heterotrophic growth on acetate and butyrate. A. Heterotrophic growth on acetate. Red: 1 (gC.L^-1^); blue: 0.5 (gC.L^-1^); purple: 0.25 (gC.L^-1^); yellow: 0.25 (gC.L^-1^); green: 0.1 (gC.L^-1^). B. Heterotrophic growth on butyrate. Red: 1 (gC.L^-1^); blue: 0.5 (gC.L^-1^); yellow: 0.25 (gC.L^-1^); green: 0.1 (gC.L^-1^). C. Autotrophic and mixotrophic growth. Red: 0.3 gC.L^-1^ acetate; blue: 0.3 gC.L^-1^ butyrate; yellow: 0.3 gC. L^-1^ acetate and 0.3 gC. L^-1^ butyrate; green: autotrophy.
(TIFF)

S11 Fig Evolution of GAP during mixotrophic and autotrophic growth. Green: autotrophy; red: mixotrophy on 0.3 gC.L^-1^ acetate; blue: mixotrophy on 0.3 gC.L^-1^ butyrate; yellow: mixotrophy on 0.3 gC.L^-1^ acetate and 0.3 gC.L^-1^ butyrate.
(TIFF)
