# Supplementary material for: Dynamic metabolic modeling of heterotrophic and mixotrophic microalgal growth on fermentative wastes
Source: PLoS Comput Biol. 2017 Jun 5;13(6):e1005590. doi: 10.1371/journal.pcbi.1005590 (PMC5476291; doi:10.1371/journal.pcbi.1005590)
Supplement: S1 File — (DOCX) [file pcbi.1005590.s001.docx]

# Metabolic network reconstruction

Since *Chlorella sorokiniana* has not been sequenced yet, no genome-scale metabolic network was possible. Using the metabolic network of eukaryotic microalgae available (*Chlorella pyrenoidosa* [1]*, Chlamydomonas reinhardtii* [2–7]*, Ostreococcus tauri* and *Ostreococcus lucimarinus* [8])*,* we deduced a core carbon metabolic network common to unicellular microalgae containing the central metabolic pathways relevant to heterotrophy and mixotrophy: photosynthesis, glycolysis, pentose phosphate pathway, citric acid cycle, oxidative phosphorylation, chlorophyll, carbohydrates, amino acids and nucleotides synthesis (Figure S2). We did not represent species-specific pathways such as the synthesis of secondary metabolites since we assumed these pathways to have negligible fluxes compared to the main pathways and thus small impact on the metabolism. Indeed, secondary metabolites have very low intracellular concentration compared to proteins, lipids, carbohydrates, DNA and RNA. The reactions of macromolecules synthesis (proteins, lipids, DNA, RNA and biomass) were lumped into generic macroscopic reactions. The description of metabolic network reconstruction is detailed in the rest of this section.

## Starting point

The metabolic network was built by incrementing the network of Kliphuis et al. (2011). This network is rather small and generic (152 metabolites, 160 reactions) and represents only the core metabolic network common to eukaryote microalgae (photosynthesis, glycolysis, pentose phosphate pathway, TCA cycle, oxidative phosphorylation, carbohydrates, lipids, protein, DNA, RNA, chlorophyll and biomass synthesis). Some modifications were performed so as to comply with the heterotrophic growth mode: addition of the acetate and butyrate pathways, and change of the functional biomass synthesis reaction to comply with the measured biomass composition [9].

## Addition of the acetate and butyrate metabolic pathways

Acetate was already present in the metabolic network of Kliphuis et al. (2011) but was lumped into a single reaction. We detailed the acetate pathway using the MetaCyc database [10] and added an external transport reaction:

| ACEext + 0.25 ATP -->ACE + 0.25 ADP + Pi  ACE + ATP <--> ACEP + ADP  ACEP + CoA <--> AcCoA + Pi |
| --- |

However, biomass growth was not possible on acetate with only this modification because the glyoxylate shunt was not present in the metabolic network, preventing the synthesis of carbohydrates and sugar precursor metabolites (E4P, G6P, GAP) necessary for biomass synthesis [11]. We thus added the glyoxylate shunt:

| AcCoA + H2O + glyoxylate --> MAL + CoA  CIT --> glyoxylate + SUC |
| --- |

The butyrate assimilation and consumption pathways were added using the MetaCyc database [10]:

| BUTYRext + 4 ATP -->BUTYR + ADP + Pi  BUTYR + AcCoA <--> ButyrylCoA + ACE  ButyrylCoA + FADH2 <--> CrotonylCoA + FAD  CrotonylCoA + H2O <--> 3-HydroxybutyrylCoA  3-HydroxybutyrylCoA + NADP <--> AceAcCoA + NADPH  AceAcCoA + CoA <--> 2 AcCoA |
| --- |

However, in eukaryote microalgae, acetate and butyrate seem to be degraded in the glyoxysome, a specialized form of a peroxisome that contains the enzymes of the glyoxylate pathway [7,12]. Hence, we added a glyoxysome compartment in the metabolic network, including the complete glyoxylate cycle, the acetate and the butyrate degradation pathways, in accordance with the ALGAGEM Model of Dal’Molin et al. (2011):

| %Butyrate  BUTYRext + 4 ATP -->BUTYR_g + 4 ADP + 4 Pi  BUTYR_g + AcCoA_g <--> ButyrylCoA_g + ACE_g  ButyrylCoA_g + O2 --> CrotonylCoA_g + H2O2_g  CrotonylCoA_g + H2O <--> 3-HydroxybutyrylCoA_g  3-HydroxybutyrylCoA_g + NAD <--> AceAcCoA_g + NADH  AceAcCoA_g + CoA_g <--> 2 AcCoA_g  2 H2O2_g --> O2 + 2 H2O  %Acetate  ACEext + 0.25 ATP -->ACE_g + 0.25 ADP + 0.25 Pi  ACE_g + ATP <--> ACEP_g + ADP  ACEP_g + CoA_g <--> AcCoA_g + Pi  %Glyoxylate cycle  AcCoA_g + H2O + OXA_g <--> CIT_g + CoA_g  AcCoA_g + H2O + glyoxylate_g <--> MAL_g + CoA_g  ISO_g <--> SUC_g + glyoxylate_g  CIT_g <--> cisAconitate_g + H2O  cisAconitate_g + H2O <--> ISO_g  MAL_g + NAD <--> OXA_g + NADH  SUC <--> SUC_g  %Oxidative phosphorylation  3.5 H + 2.5 ADP + 2.5 Pi + NADH + 0.5 O2 --> NAD + 2.5 ATP + 3.5 H2O  %Transport  -->BUTYRext  -->ACEext  SUC--> |
| --- |

## Biomass synthesis reaction

We used the measured biomass composition of *Chlorella Vulgaris* grown on 10g.L^-1^ of acetate from Liang et al. (2009) to determine the biomass synthesis reaction. However, Liang et al. only measured carbohydrates, lipids and proteins contents of the cell. We assumed that the molar ratios of DNA, RNA and chlorophyll were the same as for the autotrophic model of *Chlamydomonas reinhardtii* of Kliphuis et al. (2012), and that they were counted as proteins in Liang et al (2009). This is in agreement with the measurement reported in [2]. We thus deduce a new biomass composition (cf Section 4) and a new biomass synthesis equation:

32.687 ATP + 32.687 H2O + 0.6025 PROTEIN + 0.2641 CARB + 0.0876 PA + 0.0011 DNA + 0.0101 Chlorophyll + 0.0329 RNA --> B + 32.687 H + 32.687 ADP + 32.687 Pi

Finally, the growth-associated ATP maintenance (GAM) was replaced by the value experimentally measured by Boyle et al. (2009) for growth of *Chlamydomonas reinhardtii* on acetate. They observed 29.890 moles of ATP for 1000g of biomass. In our model, the biomass reaction yields 186g of biomass; the maintenance term is thus 5.5595 moles of ATP per mol of biomass. This maintenance term is 6 times lower than the maintenance term estimated by Kliphuis et al. (2012) in autotrophic mode. However, determining a maintenance term in autotrophic mode is challenging since the number of absorbed photons actually used in the metabolism is difficult to estimate; a fraction of the photons being dissipated through mechanisms such as non-photochemical quenching [13].

Macromolecules composition (lipids, DNA, RNA, proteins) was assumed the same as in the autotrophic model of *Chlamydomonas reinhardtii* of Kliphuis et al. (2012). The resulting metabolic network is composed of 172 reactions and 169 metabolites. The list of metabolites and their associated reactions is given in section2 and 3. Flux Coupling Analysis (FCA) revealed that no reactions of the metabolic network were blocked.

## List of reactions

|  | **Photosynthesis** |
| --- | --- |
| R1 | 8 Light + 3 ADP + 3 Pi + H + 2 cNADP --> O2 + H2O + 2 cNADPH + 3 ATP |
| R2 | CO2 + H2O + cRu15DP --> 2 cG3P |
| R3 | ATP + cG3P --> ADP + H + c13DPG |
| R4 | H + cNADPH + c13DPG <--> cNADP + Pi + cGAP |
| R5 | cGAP <--> cDHAP |
| R6 | cDHAP + cGAP <--> cF16P |
| R7 | H2O + cF16P <--> Pi + cF6P |
| R8 | cF6P + cGAP <--> cE4P + cX5P |
| R9 | H2O + cE4P + cGAP <--> Pi + cS7P |
| R10 | cGAP + cS7P <--> cR5P + cX5P |
| R11 | cX5P <--> cRu5P |
| R12 | cR5P <--> cRu5P |
| R13 | ATP + cRu5P --> ADP + H + cRu15DP |
| R14 | cGAP <--> GAP |
|  | **Glycolysis** |
| R15 | G6P <--> G1P |
| R16 | F6P <--> G6P |
| R17 | ATP + F6P --> ADP + F16P + H |
| R18 | F16P + H2O --> F6P + Pi |
| R19 | DHAP + GAP <--> F16P |
| R20 | DHAP <--> GAP |
| R21 | GAP + NAD + Pi <--> 13DPG + H + NADH |
| R22 | 13DPG + ADP <--> 3PG + ATP |
| R23 | 3PG <--> 2PG |
| R24 | 2PG <--> H2O + PEP |
| R25 | ADP + H + PEP <--> ATP + PYR |
|  | **Tricarboxylic acid cycle** |
| R26 | CoA + NAD + PYR --> AcCoA + CO2 + NADH |
| R27 | AcCoA + H2O + OXA <--> CIT + CoA + H |
| R28 | CIT + NAD <--> AKG + CO2 + NADH |
| R29 | AKG + CoA + NAD --> CO2 + NADH + SUCCoA |
| R30 | ADP + Pi + SUCCoA <--> ATP + CoA + SUC |
| R31 | FAD + SUC <--> FADH2 + FUM |
| R32 | FUM + H2O <--> MAL |
| R33 | FAD + MAL <--> FADH2 + OXA |
| R34 | ATP + CO2 + H2O + PYR --> ADP + OXA + Pi + 2 H |
| R35 | ATP + OXA --> ADP + CO2 + PEP |
| R36 | CO2 + H2O + PEP <--> H + OXA + Pi |
|  | **Pentose phosphate pathway** |
| R37 | G6P + H2O + NADP <--> 6PG + NADPH + 2 H |
| R38 | 6PG + NADP <--> CO2 + NADPH + RU5P |
| R39 | RU5P <--> R5P |
| R40 | RU5P <--> X5P |
| R41 | R5P + X5P <--> GAP + S7P |
| R42 | GAP + S7P <--> E4P + F6P |
| R43 | F6P + GAP <--> E4P + X5P |
|  | **Glycerol synthesis** |
| R44 | GLYC3P + NAD <--> DHAP + H + NADH |
|  | **N fixation** |
| R45 | H + NADH + NO3 <--> H2O + NAD + NO2 |
| R46 | 5 H + 3 NADPH + NO2 <--> NH4 + 2 H2O + 3 NADP |
|  | **S fixation** |
| R47 | ATP + SO4 --> APS + PPi |
| R48 | APS + NADH --> AMP + NAD + SO3 |
| R49 | 5 H + 3 NADPH + SO3 <--> H2S + 3 H2O + 3 NADP |
|  | **Oxidative phosphorylation** |
| R50 | 1.5 ADP + 1.5 H + 1.5 Pi + FADH2 + 0.5 O2 --> FAD + 1.5 ATP + 2.5 H2O |
| R51 | 3.5 H + 2.5 ADP + 2.5 Pi + NADH + 0.5 O2 --> NAD + 2.5 ATP + 3.5 H2O |
| R52 | H2O + PPi --> H + 2 Pi |
| R53 | AMP + ATP --> 2 ADP |
| R54 | ATP + H2O --> ADP + H + Pi + MAINT |
|  | **Amino acids and protein synthesis** |
| R55 | AKG + H + NADPH + NH4 --> GLU + H2O + NADP |
| R56 | ATP + GLU + NH4 --> ADP + GLN + H + Pi |
| R57 | AKG + GLN + H + NADPH <--> NADP + 2 GLU |
| R58 | 3PG + GLU + H2O + NAD <--> AKG + H + NADH + Pi + SER |
| R59 | SER --> NH4 + PYR |
| R60 | AcCoA + H2S + SER <--> Ace + CYS + CoA + H |
| R61 | ATP + Ace + CoA --> ADP + AcCoA + Pi |
| R62 | GLU + PYR --> AKG + ALA |
| R63 | H + THR <--> 2-oxobutan + NH4 |
| R64 | 2-oxobutan + GLU + H + NADPH + PYR <--> AKG + CO2 + H2O + ILE + NADP |
| R65 | 2 H + ALA + NADPH + PYR <--> CO2 + H2O + NADP + VAL |
| R66 | 2 PYR + AcCoA + GLU + H + NAD + NADPH <--> AKG + CoA + LEU + NADH + NADP + 2 CO2 |
| R67 | 2 PEP + ATP + E4P + NADPH --> ADP + CHO + NADP + 4 Pi |
| R68 | CHO <--> PRE |
| R69 | GLU + H + PRE <--> AKG + CO2 + H2O + PHE |
| R70 | GLU + NAD + PRE <--> AKG + CO2 + NADH + TYR |
| R71 | CHO + GLN <--> ANTH + GLU + H + PYR |
| R72 | ANTH + H + PRPP + SER <--> CO2 + GAP + PPi + TRYP + 2 H2O |
| R73 | 3 H2O + 2 NAD + ATP + GLN + PRPP --> AICAR + AKG + HIS + Pi + 2 NADH + 2 PPi + 5 H |
| R74 | GLU + OXA <--> AKG + ASP |
| R75 | ASP + ATP + GLN + H2O --> ADP + ASN + GLU + H + Pi |
| R76 | 2 ATP + 2 H2O + CO2 + GLN --> CaP + GLU + Pi + 2 ADP + 3 H |
| R77 | 2 GLU + ASP + ATP + CaP + NADH --> AKG + AMP + ARG + FUM + H2O + NAD + PPi + Pi |
| R78 | 3 H + 2 NADH + GLU <--> PRO + 2 H2O + 2 NAD |
| R79 | AKG + O2 + PRO <--> CO2 + HydPro + SUC |
| R80 | ASP + ATP + H + NADPH --> ADP + ASA + NADP + Pi |
| R81 | 2 H + ASA + GLU + NADH + PYR <--> AKG + DAP + H2O + NAD |
| R82 | DAP <--> CO2 + H + LYS |
| R83 | ASA + H + NADPH <--> HSER + NADP |
| R84 | ATP + H2O + HSER --> ADP + H + Pi + THR |
| R85 | AcCoA + CYS + H2O + HSER <--> Ace + CoA + HCYS + H + NH4 + PYR |
| R86 | HCYS + MTHF <--> H + MET + THF |
| R87 | 4.306 ATP + 3.306 H2O + 0.111 ALA + 0.092 GLY + 0.09 LEU + 0.061 VAL + 0.06 LYS + 0.056 PRO + 0.056 THR + 0.054 SER + 0.052 ARG + 0.052 GLN + 0.052 GLU + 0.047 ASN + 0.047 ASP + 0.041 PHE + 0.037 ILE + 0.03 TYR + 0.024 MET + 0.017 HIS + 0.012 CYS + 0.0090 HydPro + 0.0010 TRYP --> PROTEIN + 4.306 ADP + 4.306 Pi + 4.319 H |
| R88 | GLY + H + PYR <--> ALA + glyoxylate |
| R89 | SER + glyoxylate <--> GLY + HydPyr |
| R90 | GLY + H2O + METHF <--> SER + THF |
| R91 | GLY + NAD + THF <--> CO2 + METHF + NADH + NH4 |
| R92 | H + HydPyr + NADH <--> Glycerate + NAD |
| R93 | ATP + Glycerate --> ADP + 2 H + 3PG |
|  | **THF metabolism** |
| R94 | ATP + R5P --> AMP + H + PRPP |
| R95 | 5FTHF + H <--> H2O + MYLTHF |
| R96 | H2O + MYLTHF <--> H + N10FTHF |
| R97 | ATP + FORM + THF --> ADP + N10FTHF + Pi |
| R98 | MYLTHF + NADPH <--> METHF + NADP |
| R99 | H + METHF + NADPH <--> MTHF + NADP |
| R100 | 5FTHF + ATP + H2O --> ADP + H + N10FTHF + Pi |
| R101 | FORM + H + THF <--> H2O + N10FTHF |
| R102 | DHF + H + NADPH <--> NADP + THF |
|  | **Lipids synthesis** |
| R103 | ACP + AcCoA + H <--> AcACP + CoA |
| R104 | ATP + AcCoA + CO2 + H2O <--> ADP + H + MalCoA + Pi |
| R105 | ACP + MalCoA <--> CoA + MalACP |
| R106 | 10 H + 10 NADPH + 5 MalACP + AcACP <--> C12:0ACP + 5 ACP + 5 CO2 + 5 H2O + 10 NADP |
| R107 | 12 H + 12 NADPH + 6 MalACP + AcACP <--> C14:0ACP + 6 ACP + 6 CO2 + 6 H2O + 12 NADP |
| R108 | 14 H + 14 NADPH + 7 MalACP + AcACP <--> C16:0ACP + 7 ACP + 7 CO2 + 7 H2O + 14 NADP |
| R109 | C16:0ACP + H + NADH + O2 <--> C16:1ACP + NAD + 2 H2O |
| R110 | C16:1ACP + H + NADH + O2 <--> C16:2ACP + NAD + 2 H2O |
| R111 | C16:2ACP + H + NADH + O2 <--> C16:3ACP + NAD + 2 H2O |
| R112 | 16 H + 16 NADPH + 8 MalACP + AcACP <--> C18:0ACP + 8 ACP + 8 CO2 + 8 H2O + 16 NADP |
| R113 | C18:0ACP + H + NADH + O2 <--> C18:1ACP + NAD + 2 H2O |
| R114 | C18:1ACP + H + NADH + O2 <--> C18:2ACP + NAD + 2 H2O |
| R115 | C18:2ACP + H + NADH + O2 <--> C18:3ACP + NAD + 2 H2O |
| R116 | GLYC3P + 0.474 C16:0ACP + 0.446 C18:3ACP + 0.276 C18:2ACP + 0.253 C16:3ACP + 0.16 C18:1ACP + 0.148 C16:2ACP + 0.104 C12:0ACP + 0.051 C14:0ACP + 0.048 C18:0ACP + 0.04 C16:1ACP <--> PA + 2 ACP + 2 H |
|  | **Nucleic acids synthesis** |
| R117 | 4 ATP + 2 GLN + 2 H2O + ASP + CO2 + GLY + N10FTHF + PRPP --> AICAR + FUM + PPi + THF + 2 GLU + 4 ADP + 4 Pi + 7 H |
| R118 | ASP + CaP + H + O2 + PRPP <--> CO2 + H2O + H2O2 + PPi + Pi + UMP |
| R119 | 2 H2O2 <--> O2 + 2 H2O |
| R120 | ATP + UMP --> ADP + UDP |
| R121 | ATP + UDP <--> ADP + UTP |
| R122 | ATP + GLN + H2O + UTP --> ADP + CTP + GLU + Pi + 2 H |
| R123 | ATP + CDP <--> ADP + CTP |
| R124 | AICAR + N10FTHF <--> H2O + IMP + THF |
| R125 | ATP + H2O + IMP + NAD + NH4 --> AMP + GMP + NADH + PPi + 3 H |
| R126 | ATP + GMP --> ADP + GDP |
| R127 | ATP + GDP <--> ADP + GTP |
| R128 | ASP + GTP + IMP <--> AMP + FUM + GDP + Pi + 2 H |
| R129 | ATP + H + METHF + NADPH + UDP --> ADP + DHF + H2O + NADP + dTTP |
| R130 | ATP + CDP + H + NADPH --> ADP + H2O + NADP + dCTP |
| R131 | ATP + GDP + H + NADPH --> ADP + H2O + NADP + dGTP |
| R132 | ATP + H + NADPH <--> H2O + NADP + dATP |
| R133 | 2.372 H2O + 1.372 ATP + 0.18 dATP + 0.18 dTTP + 0.32 dCTP + 0.32 dGTP --> DNA + PPi + 1.372 ADP + 1.372 Pi + 2.372 H |
| R134 | 1.4 H2O + 0.56 ATP + 0.34 GTP + 0.16 UTP + 0.34 CTP --> 0.4 ADP + 0.4 H + 0.4 Pi + PPi + RNA |
|  | **Chlorophyll synthesis** |
| R135 | 12 H + 8 ATP + 8 GLU + 8 NADPH + 2.5 O2 --> PPorphyrin + 4 NH4 + 6 CO2 + 8 AMP + 8 NADP + 8 PPi + 13 H2O |
| R136 | 18 H + 15 NADPH + 8 ATP + 4 GAP + 4 PYR --> Phytyl-PP + 4 ADP + 4 AMP + 4 CO2 + 7 PPi + 8 H2O + 15 NADP |
| R137 | ATP + H2O + MET --> AdMET + H + PPi + Pi |
| R138 | AdHCYS + H2O <--> Ad + HCYS |
| R139 | ATP + Ad --> ADP + AMP + H |
| R140 | 4 NADPH + 2.5 O2 + 2 ATP + AdMET + Mg2 + PPorphyrin + Phytyl-PP --> AdHCYS + Chlorophyll + PPi + 2 ADP + 2 H2O + 2 Pi + 3 H + 4 NADP |
|  | **Carbohydrate synthesis** |
| R141 | G1P <--> CARB + Pi |
|  | **Biomass synthesis** |
| R142 | 5.5595 ATP + 5.5595 H2O + 0.6025 PROTEIN + 0.2641 CARB + 0.0876 PA + 0.0011 DNA + 0.0101 Chlorophyll + 0.0329 RNA --> Biomass + 5.5595 H + 5.5595 ADP + 5.5595 Pi |
|  | **Glyoxyzome** |
| R143 | BUTYR + 4 ATP <--> BUTYR_g + 4 ADP + 4 Pi |
| R144 | BUTYR_g + AcCoA_g <--> ButyrylCoA_g + ACE_g |
| R145 | ButyrylCoA_g + O2 --> CrotonylCoA_g + H2O2_g |
| R146 | CrotonylCoA_g + H2O <--> 3-HydroxybutyrylCoA_g |
| R147 | 3-HydroxybutyrylCoA_g + NAD <--> AceAcCoA_g + NADH |
| R148 | AceAcCoA_g + CoA_g <--> 2 AcCoA_g |
| R149 | 2 H2O2_g --> O2 + 2 H2O |
| R150 | ACE + 0.25 ATP<--> ACE_g + 0.25 ADP + 0.25 Pi |
| R151 | ACE_g + ATP <--> ACEP_g + ADP |
| R152 | ACEP_g + CoA_g <--> AcCoA_g + Pi |
| R153 | AcCoA_g + H2O + OXA_g <--> CIT_g + CoA_g |
| R154 | AcCoA_g + H2O + glyoxylate_g <--> MAL_g + CoA_g |
| R155 | ISO_g <--> SUC_g + glyoxylate_g |
| R156 | CIT_g <--> cisAconitate_g + H2O |
| R157 | cisAconitate_g + H2O <--> ISO_g |
| R158 | MAL_g + NAD <--> OXA_g + NADH |
| R159 | SUC <--> SUC_g |
|  | **Transport reactions** |
| R160 | # <--> CO2 |
| R161 | # <--> O2 |
| R162 | # <--> H2O |
| R163 | # <--> Pi |
| R164 | # <--> SO4 |
| R165 | # <--> NH4 |
| R166 | # <--> Mg2 |
| R167 | # --> Light |
| R168 | # <--> H |
| R169 | Biomass --> # |
| R170 | # --> BUTYR |
| R171 | # --> ACE |
| R172 | MAINT --> # |

## List of metabolites

| M1 | 13DPG | 1,3-diPhosphoglycerate |
| --- | --- | --- |
| M2 | 2-oxobutan | 2-Oxobutanoate |
| M3 | 2PG | 2-Phosphoglycerate |
| M4 | 3-HydroxybutyrylCoA_g | 3-Hydroxybutyryl-CoEnzyme A in the glyoxysome |
| M5 | 3PG | 3-Phosphoglycerate |
| M6 | 5FTHF | 5-Formyl-THF |
| M7 | 6PG | 6-Phosphogluconate |
| M8 | ACE | Acetate |
| M9 | ACEP_g | Acetyl Phosphate in the glyoxysome |
| M10 | ACE_g | Acetate of the glyoxysome |
| M11 | ACP | Acetyl-carrier protein |
| M12 | ADP | Adenosine diphosphate |
| M13 | AICAR | 5-Aminoimidazole-4-carboxamide ribonucleine |
| M14 | AKG | 2-Oxoglutarate (alpha-ketoglutarate) |
| M15 | ALA | Alanine |
| M16 | AMP | Adenosine monophosphate |
| M17 | ANTH | Anthranilate |
| M18 | APS | Adenylyl sulfate |
| M19 | ARG | Arginine |
| M20 | ASA | L-Aspartic semialdehyde |
| M21 | ASN | Asparagine |
| M22 | ASP | Aspartate |
| M23 | ATP | Adenosine triphosphate |
| M24 | AcACP | Acetyl-ACP |
| M25 | AcCoA | Acetyl-CoA |
| M26 | AcCoA_g | Acetyl-CoA of the glyoxysome |
| M27 | Ace | Acetate |
| M28 | AceAcCoA_g | AcetoAcetyl-CoEnzyme A in the glyoxysome |
| M29 | Ad | Adenosine |
| M30 | AdHCYS | S-Adenosyl-L-homocysteine |
| M31 | AdMET | S-Adenosyl-L-methionine |
| M32 | BUTYR | Butyrate |
| M33 | BUTYR_g | Butyrate in the glyoxysome |
| M34 | Biomass | Functional biomass |
| M35 | ButyrylCoA_g | Butyryl-CoEnzyme A in the glyoxysome |
| M36 | C12:0ACP | Dodecanoyl-ACP (Lauric acid) |
| M37 | C14:0ACP | Tetradecanoyl-ACP (Myristic acid) |
| M38 | C16:0ACP | Hexadecanoyl-ACP (Palmitic acid) |
| M39 | C16:1ACP | Trans-Hexadec-2-enoyl-ACP (Palmitoleic acid) |
| M40 | C16:2ACP | Hexadecadienoic acid -ACP |
| M41 | C16:3ACP | Hexadecatrienoic acid -ACP |
| M42 | C18:0ACP | Octadecanoyl-ACP (Stearic acid) |
| M43 | C18:1ACP | Cis-11-ocadecanoate-ACP (Oleic acid) |
| M44 | C18:2ACP | Linoleic acid -ACP |
| M45 | C18:3ACP | Alpha-linoleic acid -ACP |
| M46 | CARB | Carbohydrate |
| M47 | CDP | Cytidine diphosphate |
| M48 | CHO | Chorismate |
| M49 | CIT | Citrate |
| M50 | CIT_g | Citrate in the glyoxysome |
| M51 | CO2 | Carbon dioxide |
| M52 | CTP | Cytidine triphosphate |
| M53 | CYS | Cysteine |
| M54 | CaP | Carbamoyl phosphate |
| M55 | Chlorophyll | Chlorophyll |
| M56 | CoA | Coenzyme A |
| M57 | CoA_g | Coenzyme A in the glyoxysome |
| M58 | CrotonylCoA_g | Crotonul-CoEnzyme A in the glyoxysome |
| M59 | DAP | Diaminopimelate |
| M60 | DHAP | Dihydroxyacetone-P |
| M61 | DHF | Dihydrofolate |
| M62 | DNA | Deoxyribonucleic acid |
| M63 | E4P | Erythrose 4-phosphate |
| M64 | F16P | Fructose 1,6-bisphosphate |
| M65 | F6P | Fructose 6-phosphate |
| M66 | FAD | Flavin adenine dinucleotide oxidized |
| M67 | FADH2 | Flavin adenine dinucleotide reduced |
| M68 | FORM | Formic acid |
| M69 | FUM | Fumarate |
| M70 | G1P | Glucose 1-phosphate |
| M71 | G6P | Glucose 6-phosphate |
| M72 | GAP | Glyceraldehyde 3-phosphate |
| M73 | GDP | Guanosine diphosphate |
| M74 | GLN | Glutamine |
| M75 | GLU | Glutamate |
| M76 | GLY | Glycine |
| M77 | GLYC3P | Glycerol 3-phosphate |
| M78 | GMP | Guanosine monophosphate |
| M79 | GTP | Guanosine triphosphate |
| M80 | Glycerate | Glycerate |
| M81 | H | Proton |
| M82 | H2O | Water |
| M83 | H2O2 | Hydrogen peroxyde |
| M84 | H2O2_g | Hydrogen peroxyde in the glyoxyzome |
| M85 | H2S | Hydrogen sulfur |
| M86 | HCYS | Homocysteine |
| M87 | HIS | Histidine |
| M88 | HSER | Homoserine |
| M89 | HydPro | Hydroxyproline |
| M90 | HydPyr | 3-Hydroxyproline |
| M91 | ILE | Isoleucine |
| M92 | IMP | Inosine monophosphate |
| M93 | ISO_g | Isocitrate in the glyoxysome |
| M94 | LEU | Leucine |
| M95 | LYS | Lysine |
| M96 | Light | Photons |
| M97 | MAINT | Maintenance term |
| M98 | MAL | Malate |
| M99 | MAL_g | Malate in the glyoxysome |
| M100 | MET | Methionine |
| M101 | METHF | 5,10-Methylene-THF |
| M102 | MTHF | Methyl-THL |
| M103 | MYLTHF | 5,10-Methenyl-THF |
| M104 | MalACP | Malonyl-ACP |
| M105 | MalCoA | Malonyl-CoA |
| M106 | Mg2 | Magnesium |
| M107 | N10FTHF | 10-Formyl-THF |
| M108 | NAD | Nicotinamide oxidized |
| M109 | NADH | Nicotinamide reduced |
| M110 | NADP | Nicotinamidephosphate oxidized |
| M111 | NADPH | Nicotinamidephosphate reduced |
| M112 | NH4 | Ammonium |
| M113 | NO2 | Nitrite |
| M114 | NO3 | Nitrate |
| M115 | O2 | Oxygen |
| M116 | OXA | Oxaloacetate |
| M117 | OXA_g | Oxaloacetate in the glyoxosome |
| M118 | PA | Phosphatic Acid |
| M119 | PEP | Phosphoenolpyruvate |
| M120 | PHE | Phenylalanine |
| M121 | PPi | Pyrophosphate |
| M122 | PPorphyrin | Protoporphyrine |
| M123 | PRE | Prephanate |
| M124 | PRO | Proline |
| M125 | PROTEIN | Protein |
| M126 | PRPP | Phosphorybosylpyrophosphate |
| M127 | PYR | Pyruvate |
| M128 | Phytyl-PP | Phytyl-diphosphate |
| M129 | Pi | Orthophosphate |
| M130 | R5P | Ribose 5-phosphate |
| M131 | RNA | Ribonucleic acid |
| M132 | RU5P | Ribulose 5-phosphate |
| M133 | S7P | Sedoheptulose 7-phosphate |
| M134 | SER | Serine |
| M135 | SO3 | Sulphite |
| M136 | SO4 | Sulphate |
| M137 | SUC | Succinate |
| M138 | SUCCoA | Succinyl Coenzyme A |
| M139 | SUC_g | Succinate in the glyoxysome |
| M140 | THF | Tetrahydrofolate |
| M141 | THR | Threonine |
| M142 | TRYP | Tryptophan |
| M143 | TYR | Tyrosine |
| M144 | UDP | Uridine diphosphate |
| M145 | UMP | Uridine monophosphate |
| M146 | UTP | Uridine triphosphate |
| M147 | VAL | Valine |
| M148 | X5P | Xylulose 5-phosphate |
| M149 | c13DPG | chloroplast 13DPG |
| M150 | cDHAP | chloroplast DHAP |
| M151 | cE4P | chloroplast E4P |
| M152 | cF16P | chloroplast F16P |
| M153 | cF6P | chloroplast F6P |
| M154 | cG3P | chloroplast G3P |
| M155 | cGAP | chloroplast GAP |
| M156 | cNADP | chloroplast NADP |
| M157 | cNADPH | chloroplast NADPH |
| M158 | cR5P | chloroplast R5P |
| M159 | cRu15DP | chloroplast Ru15DP |
| M160 | cRu5P | chloroplast Ru5P |
| M161 | cS7P | chloroplast S7P |
| M162 | cX5P | chloroplast X5P |
| M163 | cisAconitate_g | glyxozome cisAconitate |
| M164 | dATP | Deoxy ATP |
| M165 | dCTP | Deoxy CTP |
| M166 | dGTP | Deoxy GTP |
| M167 | dTTP | Deoxy TTP |
| M168 | glyoxylate | glyxolyate |
| M169 | glyoxylate_g | glyxozome glyoxylate |

# Chemical element composition of macromolecules and metabolites allowed to accumulate (A) in the DRUM approach

|  | **C** | **H** | **O** | **N** | **P** | **S** | **Molar mass** | **Molar ratio** | **Mass ratio** |
| --- | --- | --- | --- | --- | --- | --- | --- | --- | --- |
| **Proteins** | 4.8 | 7.6 | 1.5 | 1.3 | 0 | 0.03 | 108.363 | 0.6025 | 0.351 |
| **Carbohydrates** | 6 | 10 | 5 | 0 | 0 | 0 | 162 | 0.2641 | 0.230 |
| **Lipids** | 36.3 | 63.4 | 8 | 0 | 1 | 0 | 658 | 0.0876 | 0.310 |
| **DNA** | 9.7 | 12.2 | 7 | 3.8 | 1 | 0 | 324.8 | 0.0011 | 0.002 |
| **RNA** | 9.5 | 12.8 | 8 | 3.8 | 1 | 0 | 339 | 0.0329 | 0.060 |
| **Chlorophyll** | 55 | 72 | 5 | 4 | 0 | 0 | 868 | 0.0101 | 0.047 |
| **Biomass** | 8.545 | 13.936 | 3.247 | 0.953 | 0.122 | 0.018 | 186 | - | - |
| **SUC** | 4 | 6 | 4 | 0 | 0 | 0 | 118.09 | - | - |
| **GAP** | 3 | 7 | 6 | 0 | 1 | 0 | 170.058 | - | - |

# List of reactions of the sub-networks

| **N°** | **Name** | **Reactions** | **Incoming metabolites** | **Outgoing metabolites** |
| --- | --- | --- | --- | --- |
| SN1 | Acetate & Butyrate assimilation | R143-R159, R51 | ACE, BUTYR, H_2_O, O_2_, H | SUC, H_2_O, O_2_, H |
| SN2 | Photosynthesis | R1-R14 | Light, H, H_2_O, Pi, CO_2_, O_2_ | GAP, H, H_2_O, Pi, CO_2_, O_2_ |
| SN3 | Biomass synthesis | R15-R142 | SUC, H, H_2_O, Pi, O_2_, NH_4_, SO_4_, Mg | B, H, H_2_O, Pi, CO_2_ |

# Determination of the photosynthesis kinetic

Besides light and organic carbon, nutrients are non-limiting. It was thus assumed that photosynthesis only depended on the mean light intensity within the culture (125mL Erlenmeyer flasks with 40mL of culture medium). Since incident light is relatively low (on average 135 μE.m^-2^.s^-1^), light was assumed neither saturating nor inhibiting. Photosynthesis was thus assumed proportional to the mean light intensity $I_{moy}$:

$$\alpha_{MR3}=k_{auto}*I_{moy}$$

with $k_{auto}$ (h^-1^.MB^-1^.( μE.m^-2^.s^-1^)^-1^.M) positive constant.

Attenuation of light was assumed to follow a Beer-Lambert law:

$$I\left( z \right)= I_{0}*e^{-\epsilon.z}$$

with z the depth of the Erlenmeyer (Figure 1),$I_{0}$ incident light intensity (on average 135 μE.m^-2^.s^-1^) and$\epsilon$ the light attenuation rate, mainly dependent on the chlorophyll content and the biomass concentration: $\epsilon=a*Chl+b*B+c$.

Turbidity of the medium was assumed null without the algae ($c=0$). Chlorophyll content was assumed proportional to the functional biomass since nitrogen is non-limiting [14] ($Chl = cste*x$). In this case, $\epsilon=a'*B$, with $a'$ positive constant.


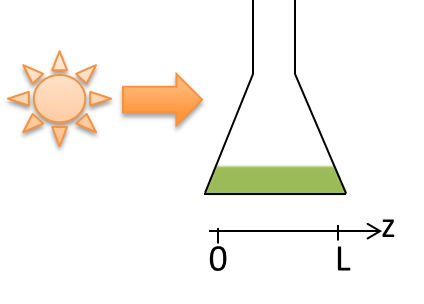


Figure 1 : Diagram of the dispositive of culture used for autotrophic and mixotrophic growth of *C sorokiniana*

Mean light intensity in the dispositive of culture is thus:

$$I_{moy}=\frac{I_{0}}{L}*\int_{0}^{L} e^{-\epsilon.z}dz= \frac{I_{0}*\left( 1-e^{-\epsilon.L} \right)}{L*\epsilon}=\frac{I_{0}*\left( 1-e^{-a^{'}.B.L} \right)}{L*a^{'}*B}$$

Photosynthesis kinetic is thus:

$$\alpha_{MR3}=k_{auto}*I_{moy}=k_{auto}*\frac{I_{0}*\left( 1-e^{-a^{'}.B.L} \right)}{a^{'}.B.L}$$

With $\gamma_{MR3}=k_{auto}*I_{0}$ and $\beta_{MR3}= L*a^{'}$, kinetic of photosynthesis equals:

$$\alpha_{MR3}=\frac{\gamma_{MR3}\left( 1-e^{\beta_{MR3}.B} \right)}{\beta_{MR3}.B}$$

# Determination of total biomass

In the DRUM framework, since there is accumulation of some intracellular metabolites, particular attention has to be drawn to the definition of biomass, which is no longer the conventional one. Biomass (B) is usually represented as an average composition of macromolecules present in the cell. With QSSA, any chemical element of substrate S ends up immediately in either biomass B or excreted products P. Since accumulation of internal metabolites is allowed in the DRUM approach, not all chemical elements from substrate S ends up in biomass B or products P; they can also be stored temporarily in the cell, under the form of metabolites A. Thus, a distinction between conventional biomass (B), which we call here functional biomass (B), and total biomass (noted X) is necessary. Total biomass X corresponds to conventional biomass B plus stored metabolites A. It can be determined thanks to a mass-balance on each chemical element using the following formulae:

$\begin{matrix} X\left( t \right)=\sum_{A} M_{A}.A\left( t \right)+M_{B}.B\left( t \right) \end{matrix}$ (1)

where$A\in\{GAP;SUC\}$, *M_A_* and *M_B_* correspond to the molar masses of *A* and *B*, *A(t)* and *B(t)* correspond to the concentration of *A* and *B* at time *t* (in M) and *X(t)* correspond to the total biomass (in g.L^-1^).

# Derived macroscopic model for heterotrophic growth

For heterotrophic growth, since succinate follows a quasi-steady state, a further reduced macroscopic model can be deduced, by merging reaction MR4 with reactions MR1 and MR2:

5.26132 H + 2 ACE+ 1.61249 O2 + 0.23756 NH4 + 0.1216 Pi + 0.00523 SO4 + 0.0024 Mg2 --> 0.24120 Biomass + 3.19844 H2O + 1.94468 CO2 (**MR’1)**

$\alpha_{MR'1} = k_{MR1}*\frac{ACE}{Ks_{MR1}+ACE}$

8.76132 H + 1 BUTYR + 2.61249 O2 + 0.23756 NH4 + 0.1216 Pi + 0.00523 SO4 + 0.0024 Mg2 --> 0.24120 Biomass + 6.69844 H2O + 1.94468 CO2 (**MR’2)**

$\alpha_{MR'2} =\frac{k_{MR2}*BUTYR}{BUTYR+\frac{k_{MR2}}{\alpha_{MR2}}*\left( \frac{BUTYR}{Sopt_{MR2}}-1 \right)^{2}}*\frac{k_{D}}{ACE+k_{D}}$

The same kinetic parameters can be used for simulation, and the fit is identical.

# Automatic detection of QSS

A quasi-steady state is reached when the evolution of the metabolite has nearly reached a steady state ($\frac{dA}{dt}\simeq0$). To detect the time interval for which the QSS hypothesis becomes valid, we chose to find the time interval from which the following property becomes satisfied during the experiment:

$\left| \frac{dA}{dt} \right|\leq c_{0}*max(\frac{dA}{dt})$

where $c_{0}$ is a scaling parameter. This criteria allows to reach satisfying results whether for SUC ($c_{0}=0.01$) (Figure 2, Figure 3) or GAP ($c_{0}=0.05$) (Figure 4). The time to reach quasi-steady state ($t_{QSS}$) was set as the time needed to reach 63% of the concentration at quasi-steady state. Because $t_{QSS}$ depends on initial biomass, the values were normalized with respect to the initial biomass of the first triplicate on acetate growth at 1gC.L^-1^.


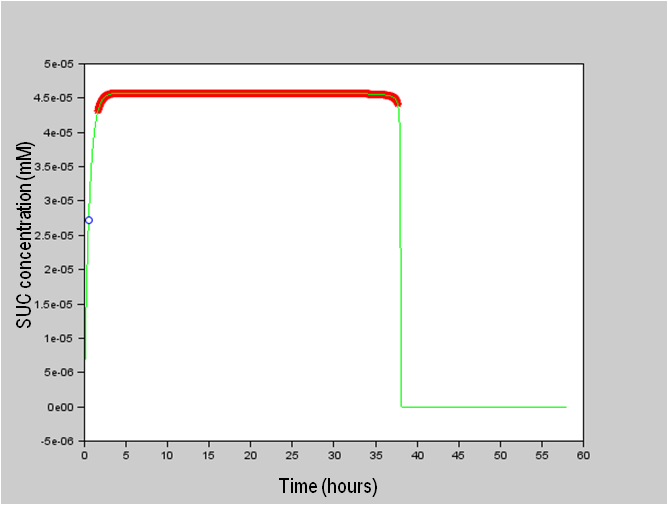


Figure 2 : Automatic detection of the quasi-steady state of succinate for heterotrophic growth on 1 gC.L^-1^ of acetate. In green: succinate concentration (mM). In red: quasi-steady state automatically detected.


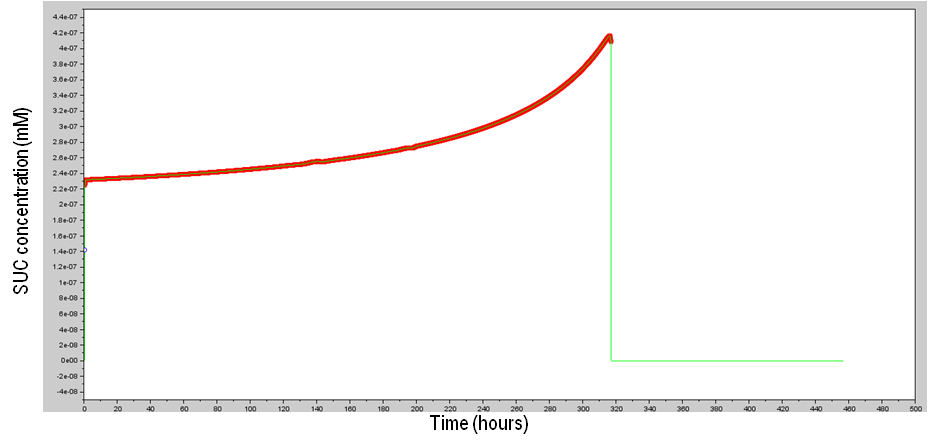


Figure 3 : Automatic detection of the quasi-steady state of succinate for heterotrophic growth on 0.1 gC.L^-1^ of butyrate. In green: succinate concentration (mM). In red: quasi-steady state automatically detected.


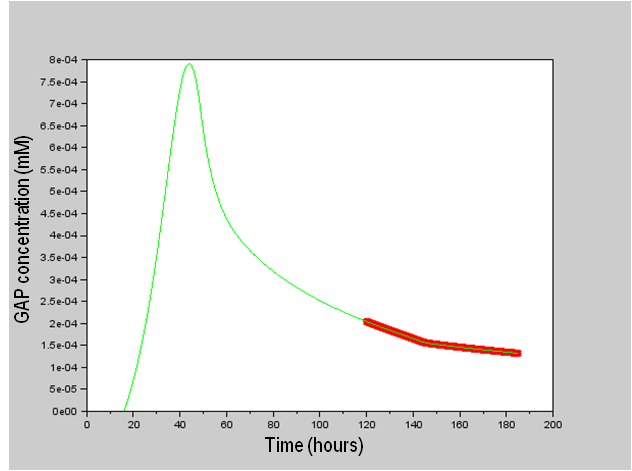


Figure 4 : Automatic detection of the quasi-steady state of GAP for mixotrophic growth on 0.1 gC.L^-1^ of acetate and 0.1 gC.L^-1^ of butyrate. In green: GAP carbon quota (mM). In red: quasi-steady state automatically detected.

To determine the confidence interval of $t_{QSS}$, we computed $t_{QSS}$ for the set of model’s parameters for which the model error was 5% higher than the minimal error. Since parameter $k_{MR4}$ had the main impact on $t_{QSS}$, $k_{MR4}$ was the only parameter whose influence was studied. We assumed that the maximal kinetic achievable in nature is five times the optimal kinetic for biomass synthesis, in order to bound $k_{MR4}$ (indeed a sensitivity analysis on this kinetic parameter (Figure 5) shows that above a threshold, the error between experimental data and the model does not further decrease).


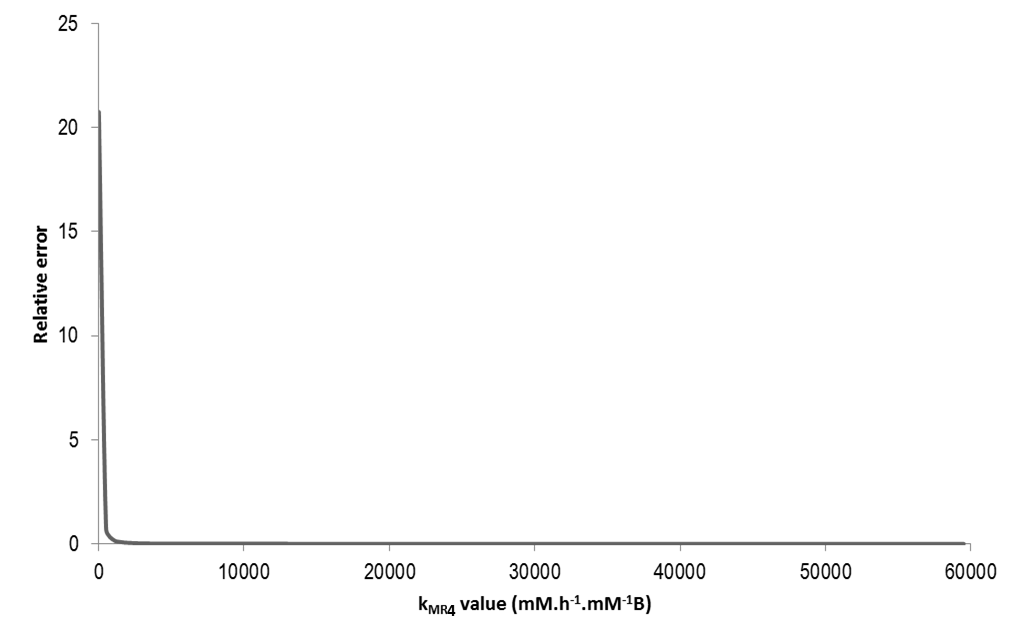


**Figure 5: Relative error function of** $\boldsymbol{k}_{\boldsymbol{MR}\boldsymbol{4}}$

$k_{MR4}$ value was varied between 0 and 200% of the value found in the optimal parameter set.

# Metabolic modeling and DRUM framework

A metabolic network can be mathematically represented by its stoichiometric matrix (*K*) of size m*r. Each row of this matrix corresponds to a metabolite, each column to a reaction and the entries are the stoichiometric coefficients. A mass-balance on the metabolism implies that:

| $\frac{dM}{dt}=\frac{d\left( \begin{aligned} S \\ C \\ P \\ B \end{aligned} \right)}{dt}=\left( \begin{aligned} K_{S} \\ K_{C} \\ K_{P} \\ K_{B} \end{aligned} \right).v.B$ | (2) |
| --- | --- |

where *M* is the metabolite concentration vector composed of substrate *S*, intracellular metabolites *C*, excreted products *P* and biomass *B*, and *v* is the vector of the kinetic rates of reactions. Because of a lack of experimental data, *v* is difficult to estimate [15]. To overcome this issue, a common hypothesis, called the quasi-steady state assumption (QSSA), assumes that the system is in a quasi-steady-state i.e. there is no accumulation of internal metabolites:

| $K_{C}.v=0, and v_{i}\geq0 if v_{i} is irreversible, i\in\{1;\ldots;r\}$ | (3) |
| --- | --- |

In any realistic large-scale metabolic model, there are more reactions than there are compounds (r > m). In other words, there are more unknown variables than equations, so there is no unique solution to (3).

Nevertheless, the set of solution is a cone, which can be described by a set of generating vectors called Elementary Flux Modes (EFMs) [16]. Any solution of (3) is a positive linear combination of the EFMs:

| $K_{C}.v=0\Leftrightarrow v=E.\alpha, \alpha\geq0$ | (4) |
| --- | --- |

with *E* the matrix of the Elementary Flux Modes and $\alpha$ a vector of positive coefficients. Elementary Flux Modes correspond to the minimal building blocks of the metabolic network [17].

The biological system can thus be viewed as a set of macroscopic reactions [18]: ${(K}_{S}.E).S\underset{\to}{\alpha}\left( K_{P}.E \right).P+\left( K_{B}.E \right).B$, with $\alpha$ the kinetic rates of the macroscopic reactions described by the stoichiometric matrix $K^{'}= \left( \begin{aligned} K_{s}.E \\ K_{P}.E \\ K_{B}.E \end{aligned} \right)$. System (2) can thus be transformed into:

| $\frac{d\left( \begin{aligned} S \\ P \\ B \end{aligned} \right)}{dt}=K^{'}.\alpha. B, \alpha\geq0$ | (4) |
| --- | --- |

Only kinetic rates $\alpha$ need to be postulated.

However, quasi-steady state assumption (no accumulation of intracellular metabolites) is a strong hypothesis, which might be inadequate for some biological systems, such as photoautotrophic metabolism of microalgae under diurnal cycles [19]. DRUM framework (Dynamic Reduction of Unbalanced Metabolism), has been proposed to handle this issue [19]. In the DRUM approach, the full metabolic network is split into sub-networks (SNs). Each SN is assumed to hold the quasi steady state assumption (QSSA). The metabolites interconnecting the sub-networks, which are named *A*, are allowed to accumulate and thus can behave dynamically. The QSSA for sub-networks relies on the presence of *i)* metabolic pathways corresponding to metabolic functions *ii)* group of reactions regulated together *iii)* different compartments in a cell (e.g., mitochondria). The metabolites (*A*) interconnecting the sub-networks are either situated at a branching point between several pathways or are end-products of metabolic pathways (e.g.: macromolecules).

DRUM framework is composed into four steps (Figure 6):

1. Find in the literature or build the metabolic network of the microorganism under study.
2. Group metabolic reactions into sub-networks assumed to follow the QSSA.
3. Reduce each sub-network to a set of macroscopic reactions using elementary mode analysis.
4. Define kinetic laws for macroscopic reactions obtained and deduce an ODE system.

*Figure was taken from* [19]*.*


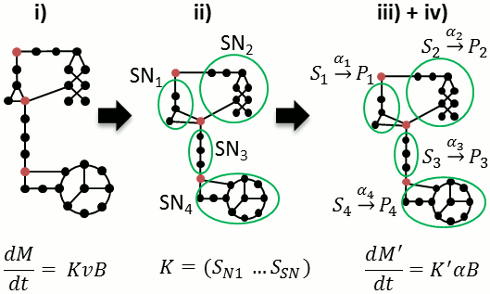


Figure Box 2: Modeling approach of DRUM decomposed into 4 steps.

For a batch operation, DRUM translates mathematically into:

| $\frac{dM^{'}}{dt}=\frac{d\left( \begin{aligned} S \\ P \\ A \\ B \end{aligned} \right)}{dt}=\left( \begin{aligned} K_{S}^{'} \\ K_{A}^{'} \\ K_{P}^{'} \\ K_{B}^{'} \end{aligned} \right).\alpha.B=K^{'}.\alpha.B$ | **(5)** |
| --- | --- |

where $M'\in\mathfrak{R}^{n_{m'}}$ is the reduced vector of compounds, consisting of substrates (*S*), products (*P*)*,* accumulating metabolites (*A*) and functional biomass (*B*). $K'\in\mathfrak{R}^{n_{m'}\times n_{E}}$ is the stoichiometric matrix of the macroscopic reactions and $\alpha\in\mathfrak{R}^{n_{E}}$ its associated rates. Note that in the full model described in step i. $K\in\mathfrak{R}^{n_{m}\times n_{r}}$,$v\in\mathfrak{R}^{n_{r}}$, while for the resulting model provided by DRUM $K'\in\mathfrak{R}^{n_{m'}\times n_{E}}$ and$\alpha\in\mathfrak{R}^{n_{E}}$, such that $n_{m}'\ll n_{m}$ and $n_{E}\ll n_{r}$.

In addition, metabolic fluxes throughout the full metabolic network can be computed thanks to:

| $v=\left( \begin{aligned} v_{SN_{1}} \\ \ldots\\ v_{SN_{k}} \end{aligned} \right)=\left( \begin{aligned} E_{SN_{1}}.\alpha_{SN_{1}} \\ \ldots\\ E_{SN_{k}}.\alpha_{SN_{k}} \end{aligned} \right)$ | **(5)** |
| --- | --- |

DRUM provides a parsimonious structure model that allows to represent the evolution of the macroscopic scale of the bioprocess as well as intracellular processes and accumulation of some metabolites.

# Computation of model prediction error

To compute the error between the model predictions and the experimental data, the following squared-error criterion was used:

| $EC=\sum_{exp\in E} \sum_{i=1}^{nb_{var}} \sum_{t\in t_{exp}} \left( \frac{Vi(t)-Vi_{exp}(t)}{Mean_{t_{exp}}\left( Vi_{exp}(t) \right)} \right)^{2}$ | **(5)** |
| --- | --- |

With *E* the set of experiments *exp* used to assess the model prediction error, $nb_{var}$ the number of experimentally measured variables *Vi* (substrates, biomass), $t_{exp}$the set of time points at which measurements were made and $Mean_{t_{exp}}\left( Vi_{exp}(t) \right)$ the mean value of the experimentally measured variable *Vi* in the experiment *exp*. The mean value is here to make dimensionless the error criterion and to avoid that variables with higher values have a higher weight in the error criterion.

# Macroscopic Bioreaction Model (MBM)

To compare the results obtained with the DRUM framework with results obtained using a classical QSSA approach, we derived a Macroscopic Bioreaction Model [20]. To do so, we computed, using *efmtool* [21], the Elementary Flux Modes of the whole metabolic network assumed at QSSA. For each substrate (light, acetate and butyrate respectively), the EFM with the best substrate/biomass yield was sought. From these EFMS, the following macroscopic reactions were derived:

5.26132 H + 2 ACE + 1.61249 O2 + 0.23756 NH4 + 0.0293297 Pi + 0.00523159 SO4 + 0.0024361 Mg2 --> 0.241198 Biomass + 1.94466 CO2 + 3.19844 H2O (**MR MBM 1)**

$$\alpha_{MR MBM1} = k_{MR1}*\frac{ACE}{Ks_{MR1}+ACE}$$

8.76132 H + 2.61249 O2 + 1 BUTYR + 0.23756 NH4 + 0.0293297 Pi + 0.00523159 SO4 + 0.0024361 Mg2 --> 0.241198 Biomass + 1.94466 CO2 + 6.69844 H2O (**MR MBM 2)**

$$\alpha_{MR MBM2} =\frac{k_{MR2}*BUTYR}{BUTYR+\frac{k_{MR2}}{\alpha_{MR2}}*\left( \frac{BUTYR}{Sopt_{MR2}}-1 \right)^{2}}*\frac{k_{D}}{ACE+k_{D}}$$

24 Light + 2.05534 CO2 + 1.30156 H2O + 0.23756 NH4 + 0.0293297 Pi + 0.00523159 SO4 + 0.0024361 Mg2 --> 0.238677 H + 0.241198 Biomass + 2.38751 O2 (**MR MBM 3)**

$$\alpha_{MR MBM3} =\frac{\gamma_{MR3.}\left( 1-e^{\beta_{MR3}.B} \right)}{\beta_{MR3}.B}$$

The parameters were re-estimated (Table 1) with the same approach used for the model derived from the DRUM framework : we minimized the squared-error between simulation and experimental measurements using the Nelder-Mead algorithm [20] (function *fminsearch* in Scilab^®^).

**Table 1: Parameters obtained by calibration of the MBM model**

| **Parameters** | **Value with DRUM** | **Definition** |
| --- | --- | --- |
| $k_{MR1}$ | 3.79*10^-1^ M.h^-1^.M B^-1^ | Maximal acetate assimilation rate |
| $Ks_{MR1}$ | 5.52*10^-5^ M | Half-saturation constant for acetate assimilation |
| $k_{MR2}$ | 3.61*10^-2^ M.h^-1^.M B^-1^ | Maximal butyrate assimilation rate |
| $\beta_{MR2}$ | 2.58*10^5^ h^-1^.M B^-1^ | Butyrate inhibition constant |
| $Sopt_{MR2}$ | 1.93*10^-5^ M | Optimal concentration for butyrate assimilation |
| $K_{D}$ | 5.39*10^-10^ M | Diauxic constant |
| $\gamma_{MR3}$ | 1.24*10^-1^ M.h^-1^.M B^-1^ | Photosynthesis kinetic parameter |
| $\beta_{MR3}$ | 1.00*10^3^ M B^-1^ | Light attenuation parameter |

Parameters different from the DRUM Model are highlighted in red.

# References

1. Yang C, Hua Q, Shimizu K. Biochem Eng J. 2000;6: 87–102.

2. Boyle NR, Morgan JA. BMC Syst Biol. 2009;3: 1–14.

3. Manichaikul A, Ghamsari L, Hom E, Chin C, Murray R, Chang R, et al. Nat Methods. 2009;6: 589–592.

4. Kliphuis A, Klok AJ, Martens DE, Lamers PP, Janssen M, Wijffels RH. J Appl Phycol. 2012;24: 253–266.

5. Chang RL, Ghamsari L, Manichaikul A, Hom EFY, Balaji S, Fu W, et al. Mol Syst Biol. 2011;7: 1–13.

6. Cogne G, Rügen M, Bockmayr A, Titica M, Dussap C-G, Cornet J-F, et al. Biotechnol Prog. 2011;27: 631–640.

7. Dal’Molin CGDO, Quek L-E, Palfreyman RW, Nielsen LK. BMC Genomics. 2011;12 Suppl 4: 1:10.

8. Krumholz EW, Yang H, Weisenhorn P, Henry CS, Libourel IGL. J Exp Bot. 2012;63: 2353–2362.

9. Liang Y, Sarkany N, Cui Y. Biotechnol Lett. 2009;31: 1043–1049.

10. Karp PD, Riley M, Paley SM, Pellegrini-Toole A. Nucleic Acids Res. 2002;30: 59–61.

11. Larhlimi A, Basler G, Grimbs S, Selbig J, Nikoloski Z. Bioinformatics. 2012;28: 502–508.

12. Perez-Garcia O, Escalante FME, de-Bashan LE, Bashan Y. Water Res. Elsevier Ltd; 2011;45: 11–36.

13. Vu TT, Stolyar SM, Pinchuk GE, Hill EA, Kucek LA, Brown RN, et al. PLoS Comput Biol. 2012;8: 1–15.

14. Mairet F, Bernard O, Lacour T, Sciandra A. Proc 18th IFAC World Congr. 2011;1: 1–6.

15. Heijnen JJ, Verheijen PJT. Biotechnol J. 2013;8: 768–775.

16. Schuster S, Dandekar T, Fell DA. Trends Biotechnol. 1999;17: 53–60.

17. Zanghellini J, Ruckerbauer DE, Hanscho M, Jungreuthmayer C. Biotechnol J. 2013;8: 1009–1016.

18. Klamt S, Stelling J. Trends Biotechnol. 2003;21: 64–69.

19. Baroukh C, Muñoz-Tamayo R, Steyer J-P, Bernard O. Vertes A, editor. PLoS One. 2014;9: e104499.

20. Provost A, Bastin G, Agathos SN, Schneider Y-J. Bioprocess Biosyst Eng. 2006;29: 349–366.

21. Terzer M, Stelling J. Bioinformatics. 2008;24: 2229–2235.
