# Supplementary material for: Dynamic metabolic modeling of heterotrophic and mixotrophic microalgal growth on fermentative wastes
Source: PLoS Comput Biol. 2017 Jun 5;13(6):e1005590. doi: 10.1371/journal.pcbi.1005590 (PMC5476291; doi:10.1371/journal.pcbi.1005590)
Supplement: S1 Table — (DOCX) [file pcbi.1005590.s004.docx]

Table S1: Comparison of existing microalgal models representing heterotrophic and/or mixotrophic growth on acetate or butyrate

| **Reference** | **Modeling type** | **Substrate** | **Metabolic Fluxes** | **Metabolite concentrations** | **Degrees of freedom** |
| --- | --- | --- | --- | --- | --- |
| (Boyle and Morgan, 2009) | Static | A, A+L | 484 | 0 | 1 |
| (Chang et al., 2011) | Static | A, A+L | 1725 | 0 | 1 |
| (Dal’Molin et al., 2011) | Static | A, A+L | 871 | 0 | 1 |
| (Chapman et al., 2015) | Static | A +L, L | 2181 | 0 | 1 |
| This article | Dynamic | A, B, A+B, A+L, B+L, A+B+L | 158 | 12 | 10 |

To compare the models, our definition of “degrees of freedom” stands for the number of numerical values needed to calibrate the model. For FBA models, degrees of freedom relate to the number of constraints needed to determine the flux distribution. Biomass compositions were not considered as degrees of freedom.

**A**: Acetate, **B**: Butyrate, **L**: Light
