# Supplementary figures and images for: Dynamic metabolic modeling of heterotrophic and mixotrophic microalgal growth on fermentative wastes
Source: PLoS Comput Biol. 2017 Jun 5;13(6):e1005590. doi: 10.1371/journal.pcbi.1005590 (PMC5476291; doi:10.1371/journal.pcbi.1005590)

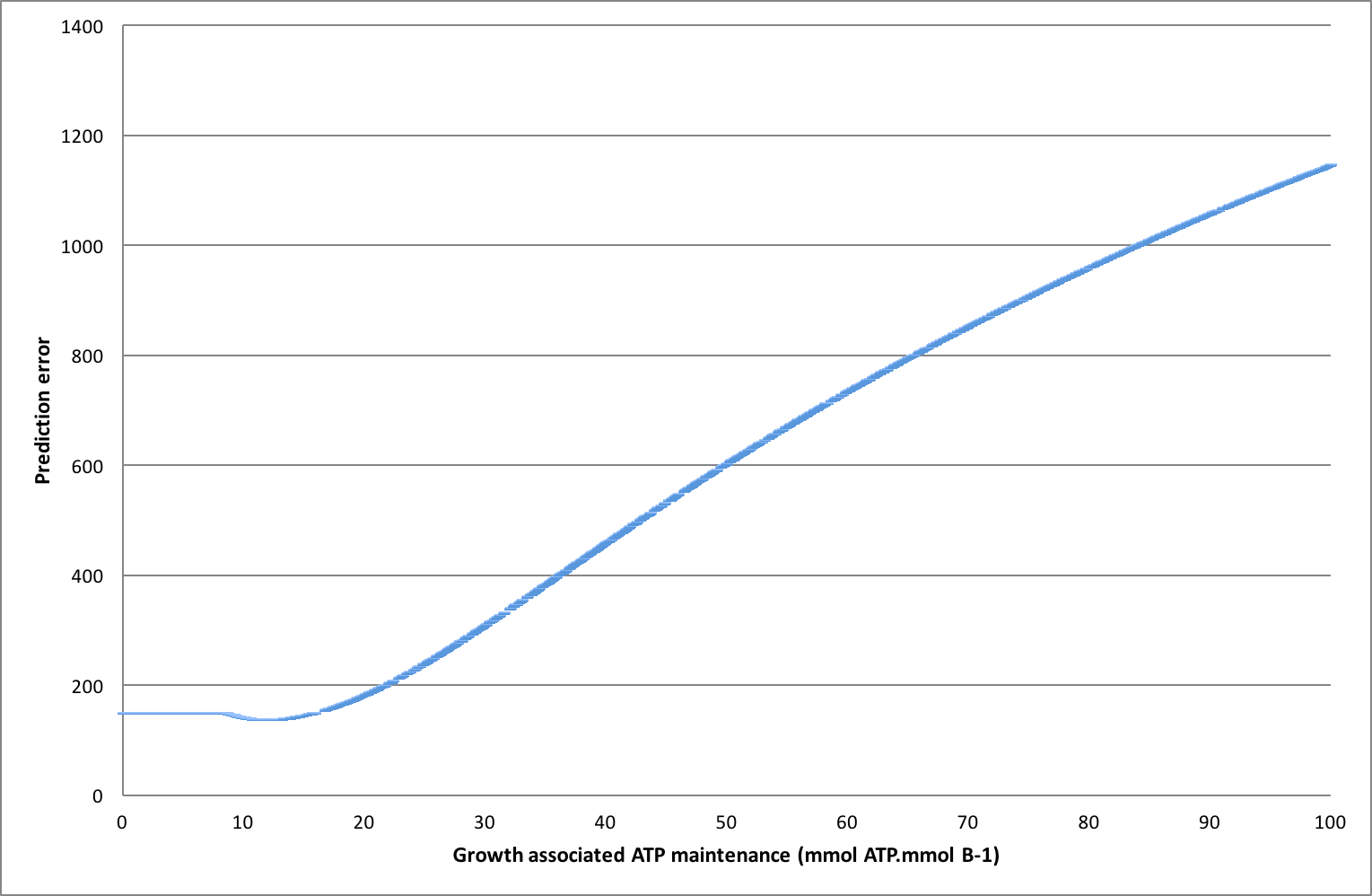

Supplement: S1 Fig — The molecular weight of biomass (B) is 186g/mol. Formula of the error criterion is given in S1 File section 8. (TIFF) [file pcbi.1005590.s005.tiff]

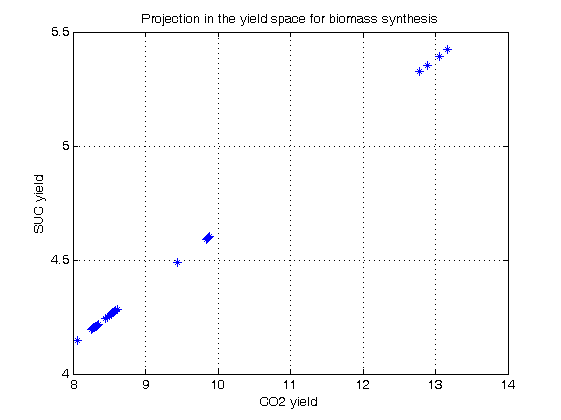

Supplement: S2 Fig — (TIFF) [file pcbi.1005590.s006.tiff]

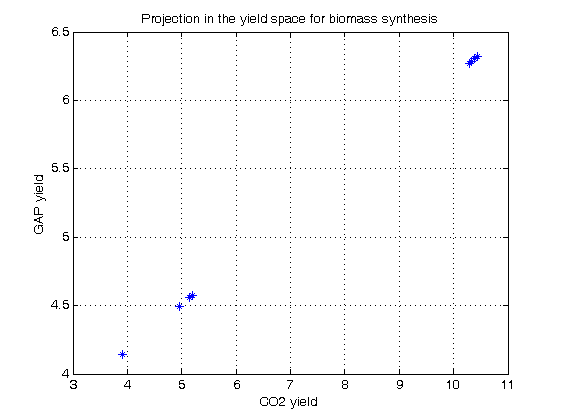

Supplement: S3 Fig — (TIFF) [file pcbi.1005590.s007.tiff]

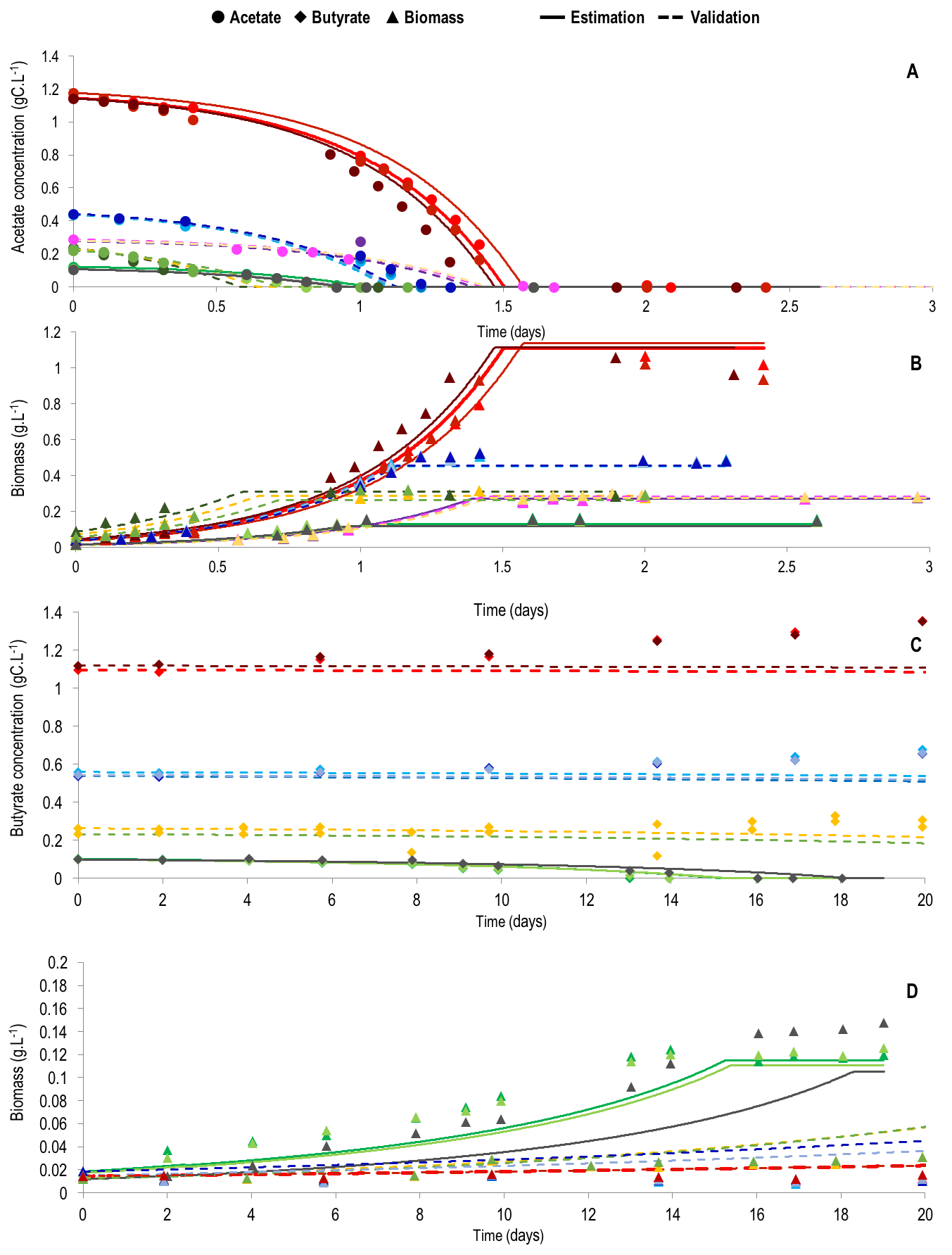

Supplement: S4 Fig — Simulations are represented by full lines (conditions used for calibration) or dashed lines (conditions used for validation). Experimental results are represented by large dots, triangles or diamonds. Shades of red: 1 gC.L-1; shades of blue: 0.5 gC.L-1; shade of purple: 0.3 gC.L-1; shade of yellow: 0.25 gC.L-1; shade of green: 0.1 gC.L-1. A. Acetate concentration (gC.L-1) for acetate growth. B. Biomass concentration (g.L-1) in acetate growing conditions. C. Butyrate concentration (gC.L-1) for butyrate growth. D. Biomass concentration (g.L-1) in butyrate growing conditions. (TIFF) [file pcbi.1005590.s008.tiff]

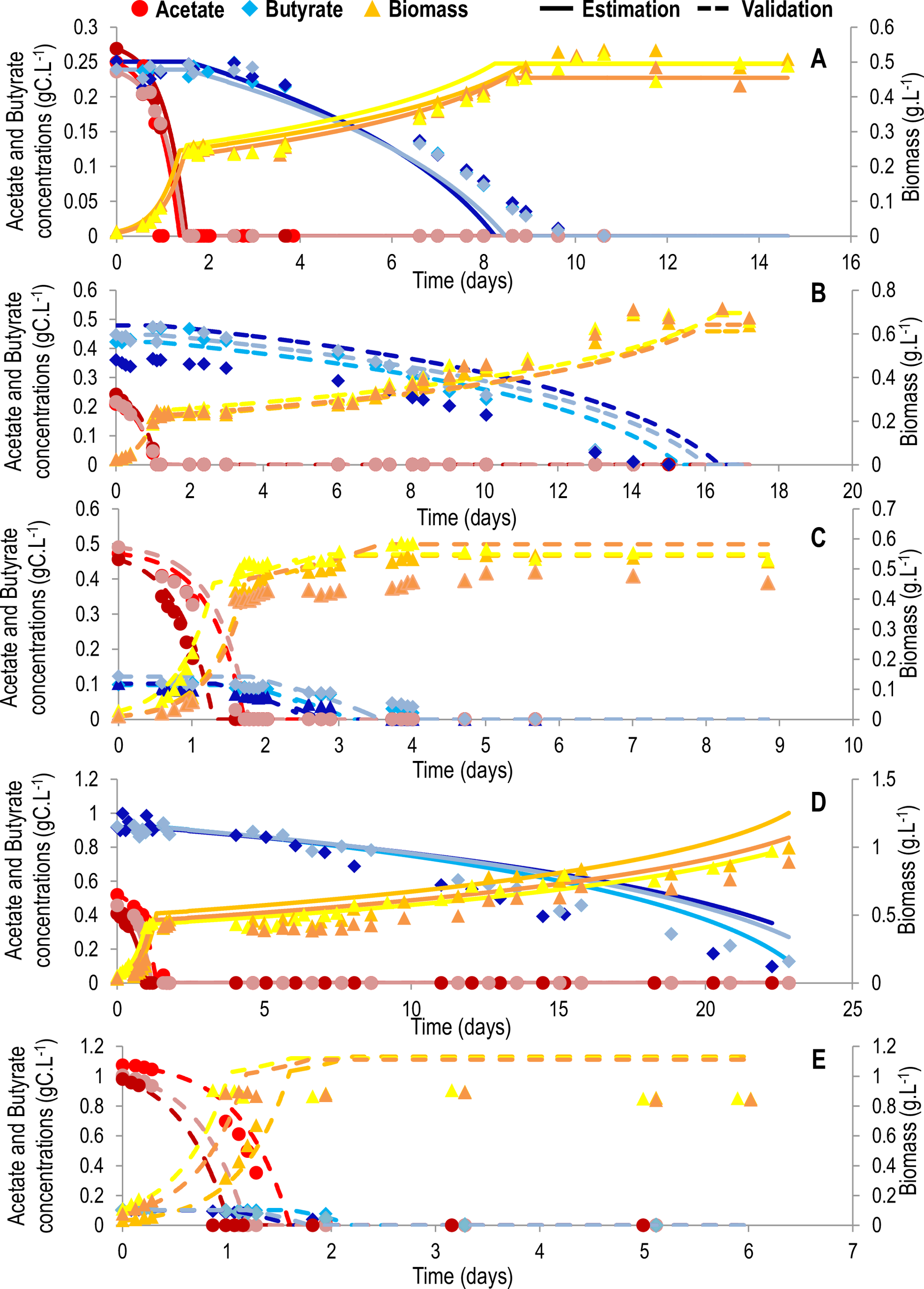

Supplement: S5 Fig — Simulations are represented by full lines (conditions used for calibration) or dashed lines (conditions used for validation). Experimental results are represented by large dots, triangles or diamonds. Shades of red: acetate (gC.L-1); shades of blue: butyrate (gC.L-1); shades of yellow: biomass (g.L-1). A. Growth on 0.25 gC.L-1 acetate and 0.25 gC.L-1 butyrate. B. Growth on 0.25 gC.L-1 acetate and 0.5 gC.L-1 butyrate. C. Growth on 0.4 gC.L-1 acetate and 0.1 gC.L-1 butyrate. D. Growth on 0.5 gC.L-1 acetate and 0.9 gC.L-1 butyrate. E. Growth on 0.9 gC.L-1 acetate and 0.1 gC.L-1 butyrate. (TIFF) [file pcbi.1005590.s009.tiff]

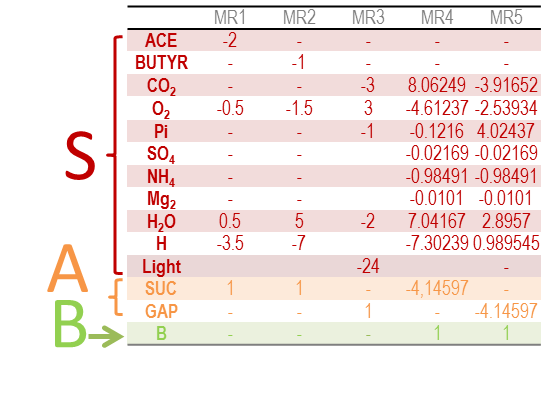

Supplement: S6 Fig — (TIFF) [file pcbi.1005590.s010.tiff]

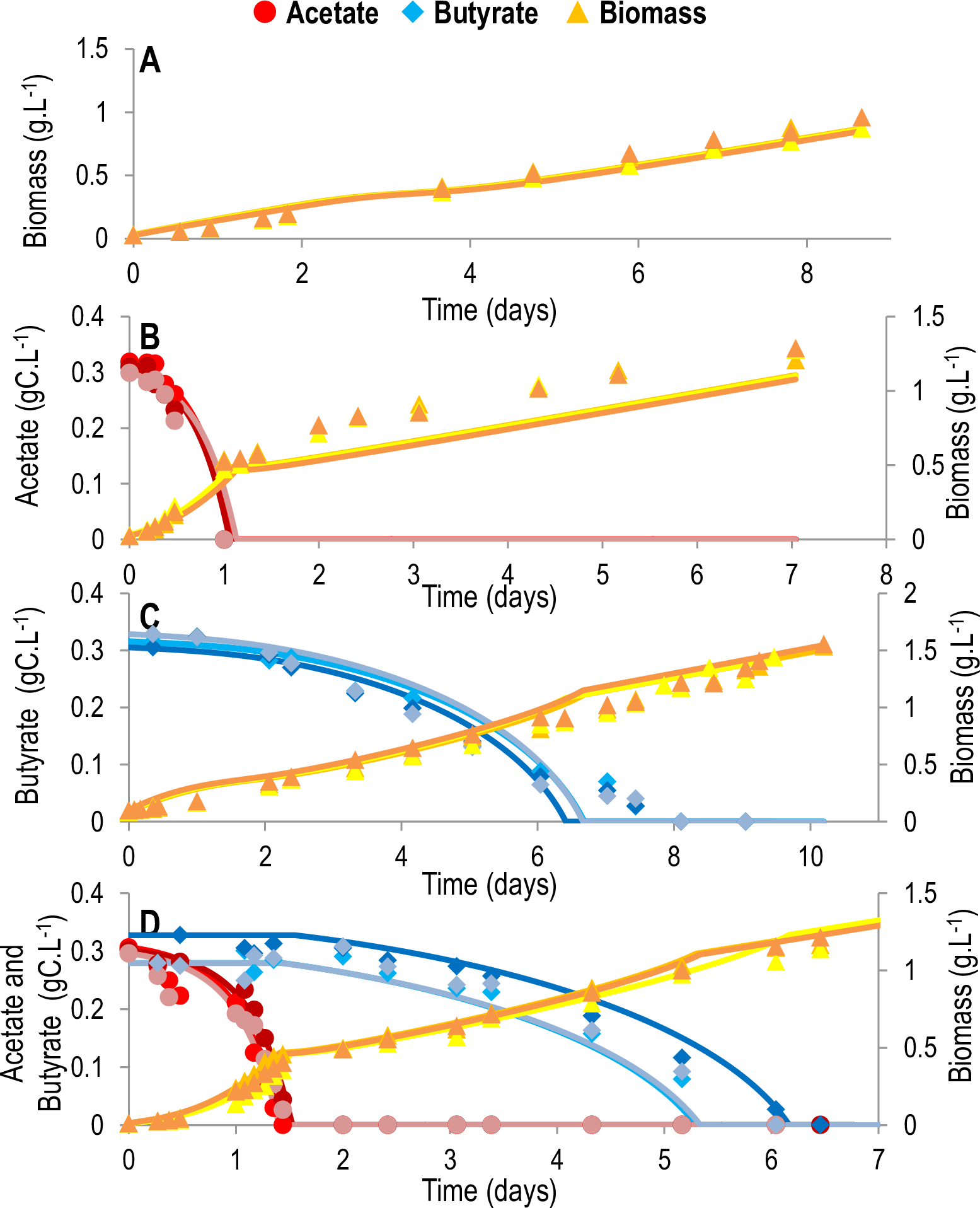

Supplement: S7 Fig — Simulations are represented by full lines (conditions used for calibration). Experimental results are represented by large dots, diamonds or triangles. Shades of red: acetate; shades of blue: butyrate; shades of yellow: biomass. A. Autotrophic growth. B. Mixotrophic growth with 0.3 gC.L-1 acetate C. Mixotrophic growth with 0.3 gC.L-1 butyrate. D. Mixotrophic growth with 0.3 gC.L-1 acetate and 0.3 gC.L-1 butyrate. (TIFF) [file pcbi.1005590.s011.tiff]

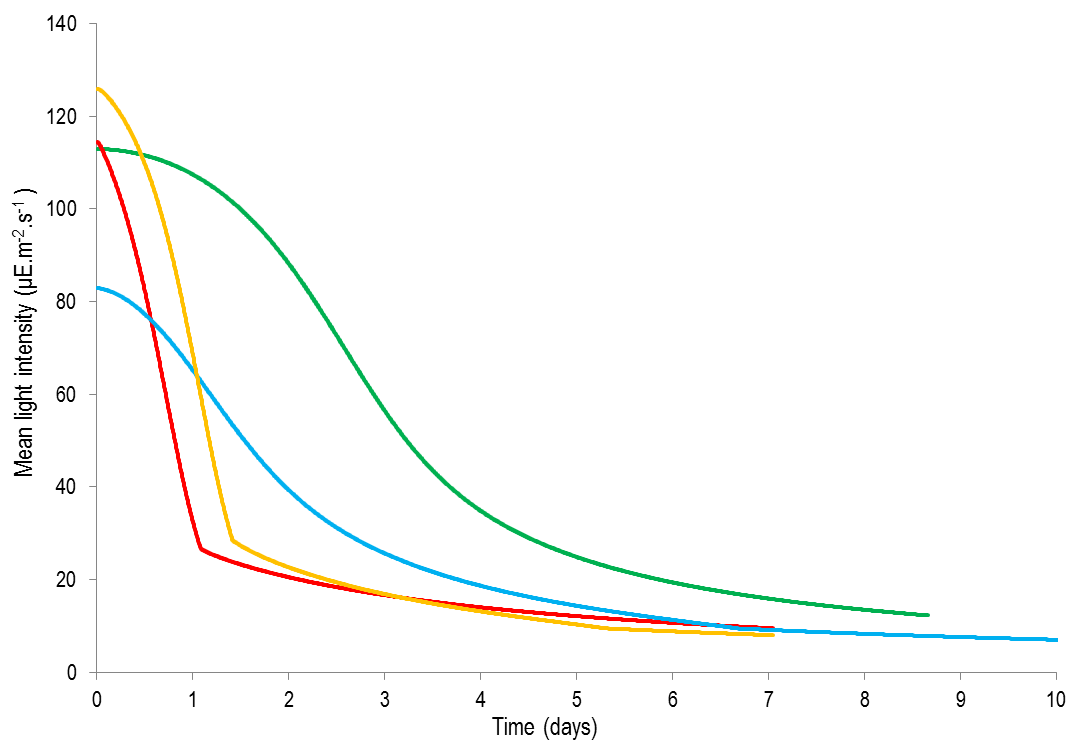

Supplement: S8 Fig — Green: Autotrophy, Red: Mixotrophy on acetate, Blue: mixotrophy on butyrate, Yellow: mixotrophy on acetate and butyrate. (TIFF) [file pcbi.1005590.s012.tiff]

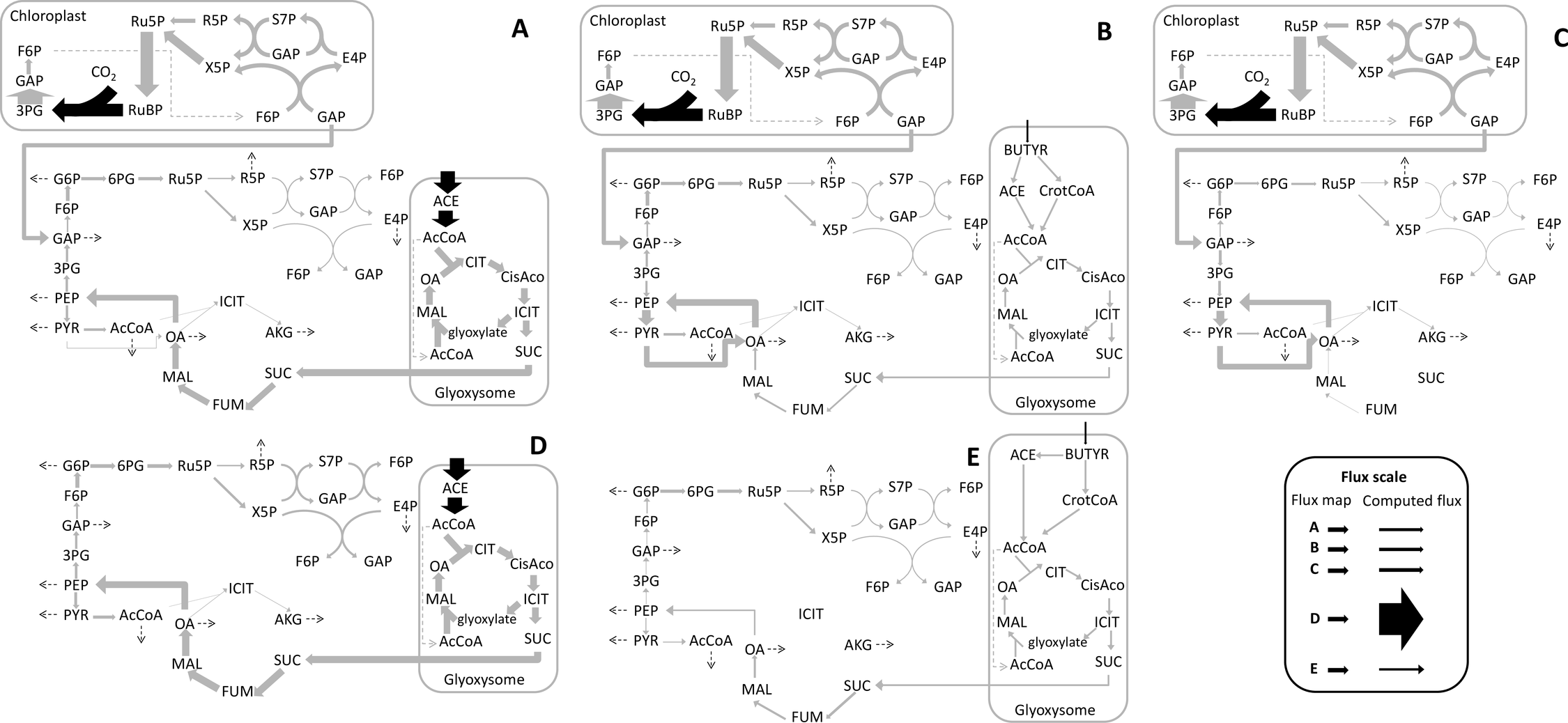

Supplement: S9 Fig — Fluxes are normalized by unit of biomass. Dashed arrows indicate fluxes related to biomass formation. Metabolic fluxes vary greatly according to substrates and growth modes. The scale for converting metabolic fluxes into arrows width is presented for each case. A. Mixotrophic growth on 0.3g.L-1 acetate. Flux maps computed at time = 0.7 days. B. Mixotrophic growth on 0.3g.L-1 butyrate. Flux maps computed at time = 5.0 days. C. Autotrophic growth. Flux maps computed at time = 5.0 days. D. Heterotrophic growth on 0.3g.L-1 acetate. Flux maps computed at time = 1.0 days. E. Heterotrophic growth on 0.1g.L-1 butyrate. Flux maps computed at time = 8.1 days. (TIFF) [file pcbi.1005590.s013.tiff]

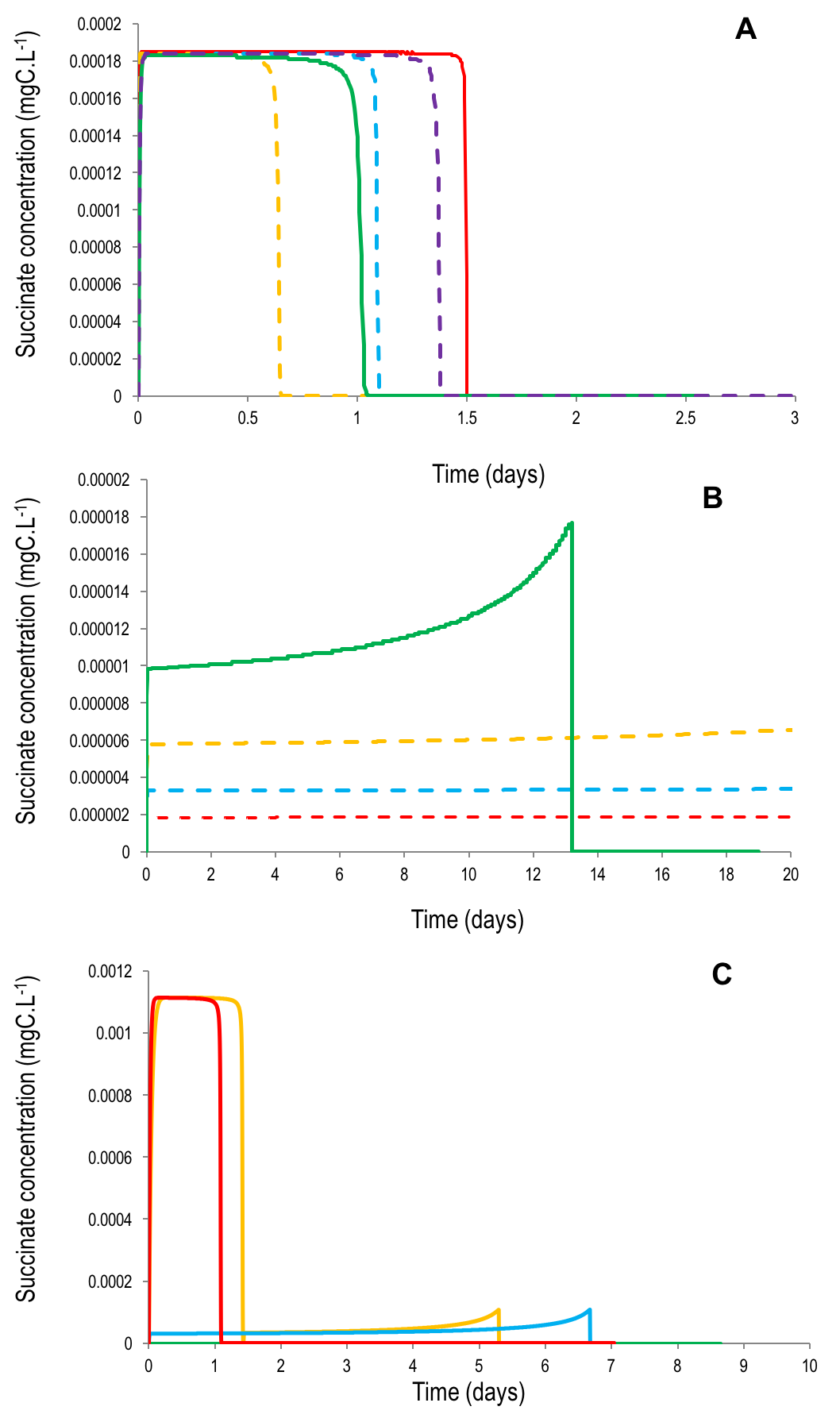

Supplement: S10 Fig — A. Heterotrophic growth on acetate. Red: 1 (gC.L-1); blue: 0.5 (gC.L-1); purple: 0.25 (gC.L-1); yellow: 0.25 (gC.L-1); green: 0.1 (gC.L-1). B. Heterotrophic growth on butyrate. Red: 1 (gC.L-1); blue: 0.5 (gC.L-1); yellow: 0.25 (gC.L-1); green: 0.1 (gC.L-1). C. Autotrophic and mixotrophic growth. Red: 0.3 gC.L-1 acetate; blue: 0.3 gC.L-1 butyrate; yellow: 0.3 gC. L-1 acetate and 0.3 gC. L-1 butyrate; green: autotrophy. (TIFF) [file pcbi.1005590.s014.tiff]

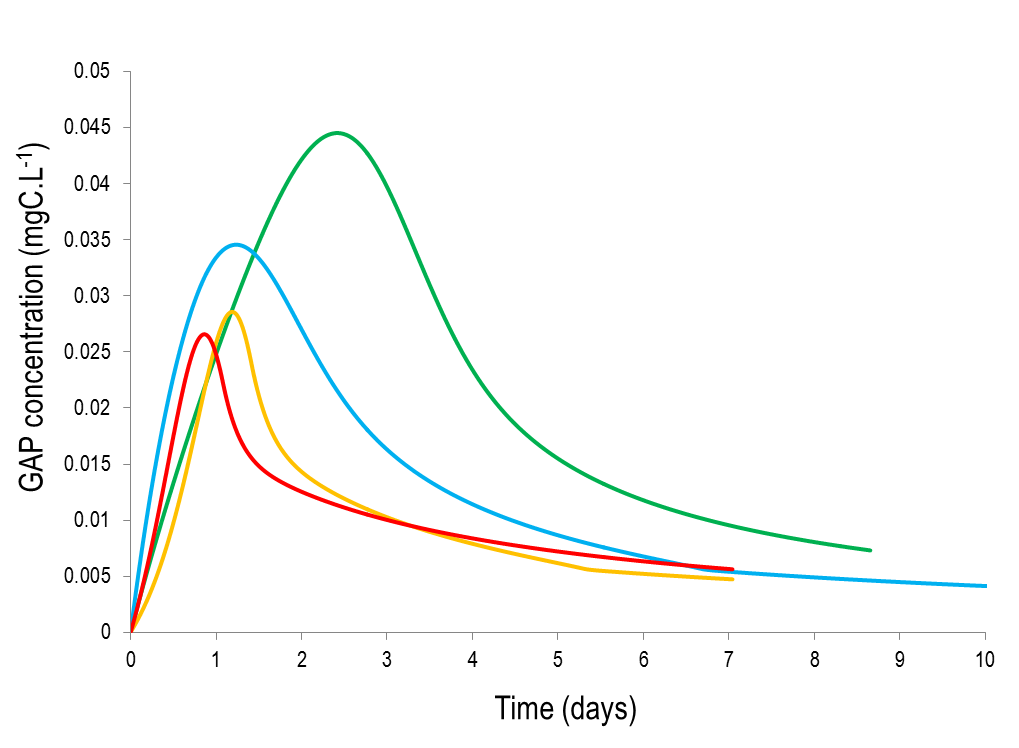

Supplement: S11 Fig — Green: autotrophy; red: mixotrophy on 0.3 gC.L-1 acetate; blue: mixotrophy on 0.3 gC.L-1 butyrate; yellow: mixotrophy on 0.3 gC.L-1 acetate and 0.3 gC.L-1 butyrate. (TIFF) [file pcbi.1005590.s015.tiff]
